# Assessment of a Helium/Argon-Generated Cold Atmospheric Plasma Device’s Safety Utilizing a Pig Model

**DOI:** 10.3390/ijms26167854

**Published:** 2025-08-14

**Authors:** Xin-Rui Zhang, Thuy-Tien Thi Trinh, Linh Le Thi Thuy, Nguyen Ngan Giang, Yong-Xun Jin, Young-Hyun Lee, Gun-Young Ahn, Boncheol Leo Goo, Kyoung-Su Jung, Hyun-Soo Hwang, Pham Ngoc Chien, Chan-Yeong Heo

**Affiliations:** 1 Department of Plastic and Reconstructive Surgery, Seoul National University Bundang Hospital, Seongnam 13620, Republic of Korea; zhangxinrui@snu.ac.kr (X.-R.Z.); jinyongxun789@snu.ac.kr (Y.-X.J.); 2 Department of Plastic and Reconstructive Surgery, College of Medicine, Seoul National University, Seoul 03080, Republic of Korea; 3 Department of Plastic and Aesthetic Surgery, Peking Union Medical College Hospital, Beijing 100730, China; 4 Korean Institute of Nonclinical Study Center, Seongnam 13620, Republic of Korea; yhlee@koreansrc.com; 5 Department of Biomedical Science, College of Medicine, Seoul National University, Seoul 03080, Republic of Korea; 6 Department of Medical Technology, Hai Phong University of Medicine and Pharmacy, Haiphong 180000, Vietnam; 7 Department of Medical Device Development, College of Medicine, Seoul National University, Seoul 03080, Republic of Korea; 8 Gowoonsesang Dermatology Clinic, Seoul 06055, Republic of Korea; doctorahn@agnesmedical.com; 9 Naeum Dermatology and Aesthetics Clinic, Seoul 04512, Republic of Korea; boncheolleogoo@agnesmedical.com (B.L.G.); jks@agnesmedical.com (K.-S.J.); 10 Agnes Medical, Seongnam 13591, Republic of Korea; ethanhwang@agnesmedical.com

**Keywords:** plasma skin therapy, dual gas plasma device, Cold Atmospheric Plasma (CAP), plasma, helium, argon

## Abstract

The PlazMagik device is a dual-gas cold atmospheric plasma (CAP) system that was developed and used for skin rejuvenation and inflammation treatment. However, preclinical evaluation and optimization of plasma parameters are crucial for guaranteeing safety. Therefore, this study was performed to evaluate the safety of the PlazMagik device under multiple parameters with different gas resources (helium (He) and argon (Ar) gases) on pig dorsal skin. After application of PlazMagik to the pig’s dorsal skin, temperature and visual assessments were observed immediately and for up to 30 days. All clinical parameters, including body weight and blood serum biochemistry, along with histopathological analysis (H&E, MT, VB, NBTC staining), were monitored pre-application and at 1, 7, 15, and 30 days post-application of the plasma device. Our results confirmed the safety of the machine at low-output energy settings, which showed gentle skin exfoliation but no tissue damage, while high-output settings led to the skin erosion effect, then developing erythema and coagulation. Ar gas resulted in more significant heat production and pathological changes than He under identical conditions. These findings emphasize the importance of the preclinical evaluation of the energy settings and gas selection on optimizing CAP system performance for safe clinical applications and appropriate application purposes.

## 1. Introduction

Recent improvements in living conditions and the increasing global elderly population have markedly intensified interest in anti-aging therapies [1,2]. Dermal health, an obvious indicator of aging, influences not just an individual’s appearance but also their self-worth and psychological well-being [2]. As a result, novel medical devices designed to delay skin aging have emerged as effective remedies. These devices frequently utilize advanced technologies such as plasma, laser treatment, microcurrents, and ultrasound to enhance skin structure and revitalize its appearance [3,4,5,6].

These technologies offer a scientifically substantiated method for enhancing skin health and longevity by targeting the fundamental causes of aging at the cellular level. However, it is essential to assess the safety and efficacy of these innovative technologies before they are implemented in clinical settings [4,7,8].

Plasma is a partially ionized gas with charged and uncharged entities. Energy processes, represented as heat or electromagnetic radiation combined with gases like argon and helium, produce plasma, which comprises charged ions and serves as an efficient conductor of electricity [9,10]. Plasma generates a dense mixture of reactive oxygen and nitrogen species (RONS), which are widely recognized as the principal oxidative mediators underlying its broad biological activity [11,12]. This property facilitates its utilization in dermatological advantages, including skin renewal, anti-inflammatory effects, and expedited wound healing, via mechanisms of proliferation, angiogenesis, and redox regulation in preclinical and early clinical studies [9,10,13,14].

Nevertheless, plasma intensity requires careful regulation to avert unintended biological effects. Cold atmospheric plasma (CAP) devices produce physical plasma at temperatures comparable to tissue under atmospheric conditions. This technique has been extensively researched for various medical applications and has emerged as a groundbreaking therapeutic innovation in dermatology [7].

Recent findings indicate that low levels of plasma might boost the skin’s natural antioxidant systems, leading to further research on using plasma to manage oxidation processes in wound management [15]. Additionally, plasma has been investigated for its potential in enhancing intracellular delivery and transdermal drug administration [16,17]. Medically applied cold atmospheric plasma (CAP) is currently generated using two main configurations: plasma jets and dielectric barrier discharges (DBDs). Plasma jet systems require a carrier gas—typically helium or argon—to maintain the plasma plume, whereas DBD devices operate by igniting plasma directly in ambient air without an external gas source [18]. Notably, cold atmospheric plasma jet (CAP-jet) demonstrates promising outcomes in dermatology, particularly in wound healing and antimicrobial efficacy [14,19]. The PlazMagik device, a CAP-jet system, was previously demonstrated for its efficacy in the improvement of photoaging skin using a mouse model [20,21]. Consequently, this study further examined the safety of the PlazMagik device in a porcine model to furnish safety data for prospective clinical applications.

Swine models showed that the anatomical and physiological structures of swine skin were similar to those of human skin [22,23]. These similarities include the thickness of the epidermis, the ratios of thickness between the epidermis and the dermis, and the number of cell layers in the stratum corneum [24,25]. The histological structure of epidermal keratins, dermal collagen, vimentin, and fibronectin is analogous to that of humans [25,26]. Significantly, pig wounds generally heal through epithelialization, similarly to human wounds, while wounds in mice, rabbits, and dogs predominantly heal through contraction, offering the porcine model as the most appropriate for cutaneous studies [27]. A thorough evaluation of several wound healing therapies in different animal models revealed that the swine model exhibited a 78% concordance with humans, as previously reported [22]. Consequently, the porcine skin model in the current study presents a meaningful approach for conducting dermal studies to evaluate the safety and efficacy of medical devices intended for dermal application.

The absence of consistent, standardized protocols for CAP application in dermatology has significantly hindered both reproducibility and efficacy assessment. CAP’s performance is modulated by several adjustable variables—including gas composition, flow rate, power input, electrode design, and treatment duration—which vary widely across devices and studies [10,14,28,29]. This variation complicates inter-study comparisons and the determination of an optimal treatment regimen.

However, a major step forward has emerged: the German Institute for Standardization issued DIN SPEC 91315:2025-07, a pre-standard defining general requirements and test methods for medical CAP sources, covering both physical characteristics and biological safety evaluations [30]. To translate standardized protocols into clinical benefit, carefully controlled trials are essential. These trials should identify optimal treatment durations and parameter configurations tailored to specific skin conditions while maintaining patient comfort and minimizing adverse effects. Through this approach, the field can advance toward safe, effective, and harmonized CAP-based dermatotherapies.

To fill the existing gap in standardized testing, this study evaluates the safety of the PlazMagik device in a porcine model, offering critical insights before progressing to human clinical trials. Specifically, we focus on assessing the effects and safety of a dual-gas plasma device, PlazMagik, on a pig model under varying gas resources and parameter conditions. Throughout the study, we monitor key indicators, including pig body weight, blood chemical parameters, and skin histopathology, to comprehensively understand the device’s impact.

## 2. Results

### 2.1. Experimental Schematic and Assessment of Clinical Value Following Plasma Irradiation

**Figure 1:**
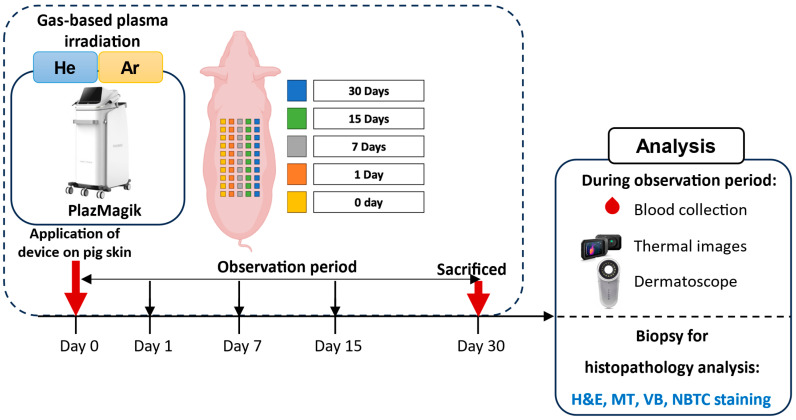
Schematic of the experimental setup used to evaluate the safety of the He/Ar-CAP device.

To evaluate the novel plasma irradiation device’s safety with different gas setting modes, we have designed an experiment as depicted in Figure 1. A total of six animals were irradiated with nine different conditions of the device (Table 1), and the two gas resources were helium and argon. Body weight was measured on test days (day 0, day 1, day 7, day 15, day 30). During the test period (30 days), body weight was maintained within the normal range without any abnormalities (Table S1, Figure S1). 

### 2.2. Measurement of Temperature Change on Pig Skin Surface Immediately After Plasma Irradiation

**Figure 2:**
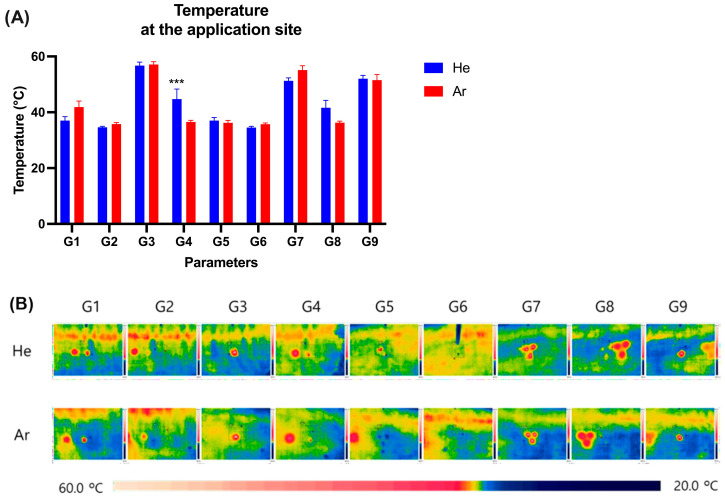
The immediate temperature of the skin surface after the plasma irradiation. The temperature at the application sites was measured as shown in graph (**A**) and observed as thermal images (**B**). Data was displayed as mean ± SD of three investigations (n = 3). Significance was calculated by two-way ANOVA followed by Sidak’s multiple comparisons test. Significance levels were set at *** *p* < 0.001. G1–G9: 9 different parameter conditions of the plasma device; He: Helium gas resource, Ar: Argon gas resource.

The temperature at the irradiation sites was assessed immediately after treatment using a thermal camera, which showed the overall temperature was below 60 °C (Figure 2A, Table S2).

Both He and Ar gas resources under conditions G1, G2, G5, G6, and G8 showed the temperature at 34.5 to 42 °C, confirming no dramatic increase in skin surface temperature due to irradiation.

Meanwhile, the temperatures were higher under the remaining conditions, G3, G4, G7, and G9, with both gas resources. The temperature was at 56.7 ± 2.2, 44.7 ± 6.3, 51.3 ± 1.8, and 52.0 ± 2.1 °C, respectively, while using He gas, whereas Ar gas under conditions G3, G7, and G9 were observed at 57.1 ± 1.8, 55.1 ± 2.7, and 51.5 ± 3.5 °C, respectively (Figure 2).

### 2.3. Dermatoscopic Observation of Skin Damage and Healing Process Following Plasma Device Application

**Figure 3:**
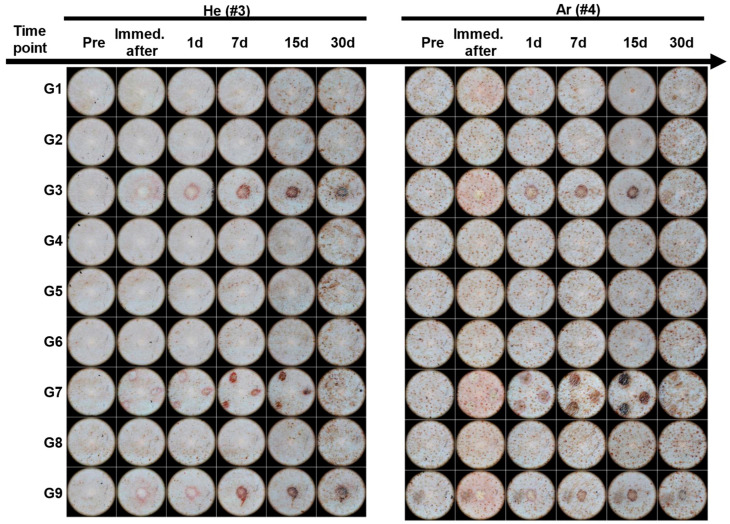
Observation of skin changes using dermatoscope after plasma irradiation (#3: Animal No. 3, #4: Animal No. 4). G1–G9: 9 different parameter conditions of the plasma device; He: Helium gas resource, Ar: Argon gas resource; 1 d: 1 day, 7 d: 7 days, 15 d: 15 days, 30 d: 30 days post-irradiation day.

We monitored the effects of CAP treatment on skin morphology and healing progress for 30 days after treatment. As shown in Figure 3, no changes in the skin surface such as erythema, pigmentation, papules, purpura, nodules, plaques, wheals, scales, and erosions were observed under conditions G1, G2, G4, and G6 of He gas and conditions G2, G4, G5, G6, and G8 of Ar gas.

Under the G3, G7, and G9 conditions of both gases, blisters due to burns and erythema in the surrounding area were observed after application, and after 7 days, a scab was formed, and healing was observed.

For He gas, under the G4 and G8 conditions, the skin surfaces of animals 1 and 2 changed as shown in Figure S2, and in animal 3 no changes in the skin surface were observed (Figure 3).

Meanwhile, under the Ar gas conditions G1, G3, G7, and G9, damage to the skin in the irradiated area and a decrease in the skin layer height were observed immediately after application, resulting in a fine appearance. Erythema in the surrounding area was observed on the first day, and a healing pattern was observed with the formation of a scab after 7 days (Figure S2).

### 2.4. Blood Serum Biochemistry Measurements

**Figure 4:**
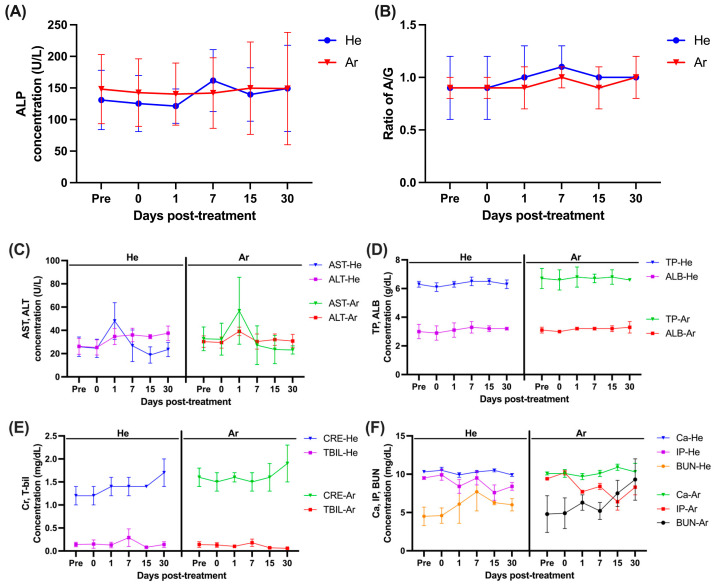
Blood serum biochemistry measurements. The blood serum was collected at the indicated time points to check for the level of alkaline phosphatase (ALP) (**A**); A/G Ratio (**B**); aspartate transaminase (AST) and alanine aminotransferase (ALT) (**C**); total protein (TP) and albumin (ALB) (**D**); creatinine (Cr) and total bilirubin (T-Bil) (**E**); calcium (Ca), inorganic phosphorus (IP), and blood urea nitrogen (BUN) (**F**). Data displayed as mean ± SD (*n* = 3). For comparison of the difference between the gas resources in each treatment condition, statistical analysis was assessed by two-way ANOVA analyses followed by a Sidak’s multiple comparisons test (asterisk in black). One-way ANOVA followed by Dunnett’s multiple comparisons test was performed to determine the difference between the control group and treatment conditions within each gas resource (asterisk in blue (Ar)/red (He)). He: Helium gas resource, Ar: Argon gas resource.

In order to observe hematological changes after treatment with the PlazMagik equipment, blood was collected on day 0, day 1, day 7, day 15, and day 30 post-treatment. The blood serum was separated and used to check for the level of creatinine (Cr), calcium (Ca), the blood urea nitrogen (BUN), inorganic phosphorus (IP), total protein (TP), albumin (ALB), A/G ratio, total bilirubin (T-bil), alanine aminotransferase (ALT), aspartate transaminase (AST), and alkaline phosphatase (ALP) (Figure 4, Tables S3 and S4). Our data showed that the AST level in animals No. 1~3 increased from 25.9 U/L at the time before treatment to 48.0 U/L on day 1 post-treatment and then turned back to the normal range on day 7 (Table S3), and the AST level in animals No. 4~6 also increased from 32.7 at pretreatment to 56.8 U/L at 1 day post-treatment and returned to the normal range after 1 week (Figure 4C, Table S4). During the observation period, although ALT-He exhibited a modest increase, this change did not reach statistical significance (*p* > 0.05) and remained within the normal reference range (Figure 4C). Meanwhile, the other criteria values were measured within the normal range without abnormal findings (Figure 4A–F).

### 2.5. Evaluation of Inflammatory Cell Infiltration and Epidermal Proliferation of Skin After Plasma Irradiation

**Figure 5:**
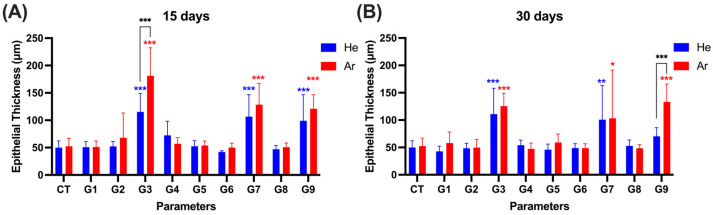

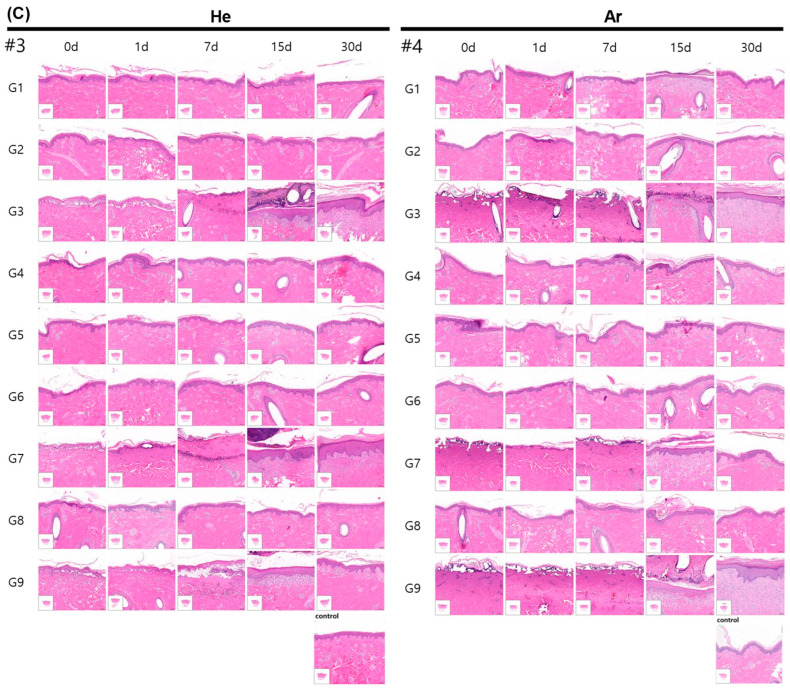
Histopathology analysis by H&E staining. After application with the device’s indication parameters, the thickness of epithelial tissue at the application site was evaluated on day 15 (**A**) and day 30 (**B**). Skin biopsy was collected and analyzed by H&E staining (#3: Animal No. 3, #4: Animal No. 4) (**C**). G1–G9: 9 different parameter conditions of the plasma device; He: Helium gas resource, Ar: Argon gas resource; 0 d: 0 day (on the treatment day), 1 d: 1 day, 7 d: 7 days, 15 d: 15 days, 30 d: 30 days post-irradiation day. Significance levels were set at * *p* < 0.05, ** *p* < 0.01, *** *p* < 0.001, data displayed as mean ± SD (*n* = 3). For comparison of the difference between the gas resources in each treatment condition, statistical analysis was assessed by two-way ANOVA analyses followed by a Sidak’s multiple comparisons test (asterisk in black). One-way ANOVA followed by Dunnett’s multiple comparisons test was performed to determine the difference between the control group and treatment conditions within each gas resource (asterisk in blue (Ar)/red (He)).

We evaluated inflammatory cell infiltration, epidermal proliferation, and dermal changes at the application sites using tissues stained with hematoxylin and eosin (H&E) (Figure 5 and Figure S3, and Table S5).

As shown in Figure 5 and Figure S3, no adverse reactions were observed under He gas conditions G1, G2, G5, and G6, as the tissue morphology remained similar to the control tissue. However, under conditions G3, G7, and G9, basal and dermal layer separation, epithelial cell shrinkage, vacuolization, and intracellular nuclear deformation were observed at 1 day post-treatment. On the 7th day, partial epithelial shedding and inflammatory cell infiltration occurred. By days 15 and 30, re-epithelialization and thickened epithelial tissue were observed (Figure 5A,B). Epithelial thickness decreased from day 15 to day 30 (G3: 115.29 ± 33.61 μm to 111.02 ± 47.30 μm; G7: 106.51 ± 40.32 μm to 100.66 ± 62.38 μm; G9: 99.10 ± 47.57 μm to 70.37 ± 15.70 μm) but remained elevated compared to the control group (49.92 ± 12.21 μm) (Table S5).

Under He gas conditions G4 and G8, no adverse effects were observed in animal No. 3. However, animals 1 and 2 exhibited basal and dermal layer separation, epithelial cell shrinkage, vacuolization, and nuclear deformation immediately and 1 day post-treatment. Epithelial thickness for G4 decreased from 72.31 ± 25.87 μm on day 15 to 54.00 ± 9.44 μm on day 30, while G8 showed no difference from the control group, with 47.04 ± 6.97 μm on day 15 and 52.79 ± 10.79 μm on day 30.

For Ar gas, no adverse reactions were observed under conditions G2, G4, G5, G6, and G8. Under condition G1, epithelial cell shrinkage, vacuolization, and nuclear deformation were observed immediately after treatment (Figure 5C). By days 15 and 30, re-epithelialization was complete, with epithelial thickness of 51.18 ± 10.73 μm and 58.07 ± 20.06 μm, respectively, which was comparable to the control group (52.33 ± 14.65 μm) (Table S5).

Under conditions G3, G7, and G9 using Ar gas, epithelial tissue deformation and loss were observed immediately after treatment, followed by re-epithelialization and hyperproliferation on days 15 and 30. Epithelial thickness decreased from day 15 to day 30 (G3: 181.16 ± 51.31 μm to 125.47 ± 23.57 μm; G7: 128.43 ± 38.74 μm to 103.26 ± 88.26 μm; G9: 120.91 ± 25.60 μm to 133.29 ± 32.60 μm) but remained thicker than the control group (Figure 5A,B, Table S5).

Our results showed that He treatments G1, G2, G5, and G6, as well as Ar treatments G2, G4, G5, G6, and G8, caused little to no damage, and the tissue looked similar to the control samples, showing that the tissue can tolerate mild plasma exposure well. These conditions produced negligible damage and satisfactory recovery, indicating nominal safety and therapeutic potential.

### 2.6. Evaluation of Skin Proliferation After Plasma Irradiation

**Figure 6:**
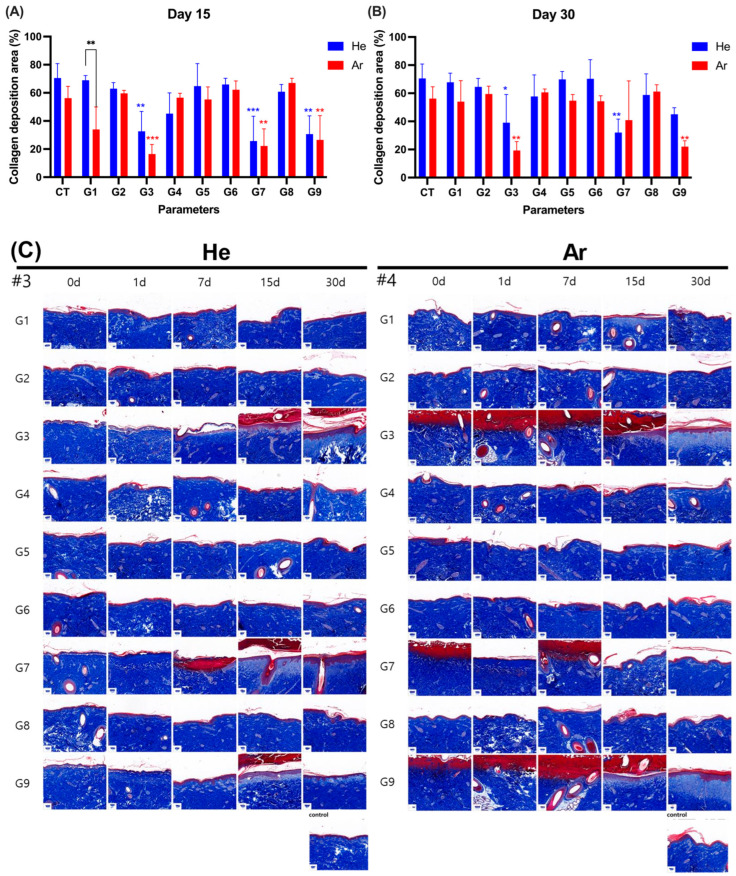
Observation of collagen deposition rate at the application site. Collagen deposition rate (%) was evaluated at 15 days (**A**) and 30 days (**B**) post-application of device at indicated parameter using Masson’s trichrome staining (#3: Animal No. 3, #4: Animal No. 4) (**C**). Data was displayed as mean ± SD. G1–G9: 9 different parameter conditions of the plasma device; He: Helium gas resource, Ar: Argon gas resource; 0 d: 0 day (on the treatment day), 1 d: 1 day, 7 d: 7 days, 15 d: 15 days, 30 d: 30 days post-irradiation day. Significance levels were set at * *p* < 0.05, ** *p* < 0.01, *** *p* < 0.001, data displayed as mean ± SD (*n* = 3). For comparison of the difference between the gas resources in each treatment condition, statistical analysis was assessed by two-way ANOVA analyses followed by a Sidak’s multiple comparisons test (asterisk in black). One-way ANOVA followed by Dunnett’s multiple comparisons test was performed to determine the difference between the control group and treatment conditions within each gas resource (asterisk in blue (Ar)/red (He)).

To observe damage to collagen fibers, tissues stained with Masson’s trichrome (MT) were photographed and observed using a slide scanner (Figure 6 and Figure S4, and Table S6).

Under He gas conditions G1, G2, G5, and G6, no deformation or damage were observed compared to the control tissue (Figure 6C and Figure S4) (*p* > 0.05). In contrast, conditions G3, G7, and G9 showed the deformation of collagen fibers on day 1, followed by inflammatory cell infiltration on day 7 and collagen reorganization by day 15. By day 30, the collagen fibers displayed increased ratios and greater maturity (e.g., G3: 32.60 ± 14.21% to 39.05 ± 20.05%; G4: 45.22 ± 14.71% to 57.67 ± 15.40%; G7: 25.69 ± 17.65% to 32.03 ± 9.60%; G9: 30.65 ± 12.95% to 45.06 ± 4.66%) (Table S6). However, these ratios remained lower than those in the control group (75.55 ± 10.27%). Animal No. 3 exhibited no significant differences under G4 and G8, whereas animals No. 1 and 2 displayed tissue recovery through deformation and subsequent reorganization of collagen fibers (Figure S4).

For Ar gas-based plasma irradiation, no noticeable damage was observed in the G2, G4, G5, G6, and G8 groups compared to the control group (56.19 ± 8.44%) (Figure 6A,B). However, collagen degradation was observed under conditions G1, G3, G7, and G9, with reorganization becoming evident by day 15 and an increase in collagen fiber maturity by day 30. Despite this, the collagen fiber ratios under these conditions remained lower than the control group. Specific changes included increases in collagen fiber ratios from day 15 to day 30 in G3 (16.47 ± 6.82% to 19.30 ± 6.35%), G7 (22.17 ± 12.21% to 40.86 ± 27.95%), and G9 (26.55 ± 17.28% to 22.03 ± 4.19%). G1 exhibited a reduced collagen fiber ratio on day 15 (33.93 ± 16.03%), but by day 30, it improved to align with the control group (54.05 ± 14.88%) (Table S6).

### 2.7. Evaluation of Skin Elasticity Post-Irradiation

**Figure 7:**
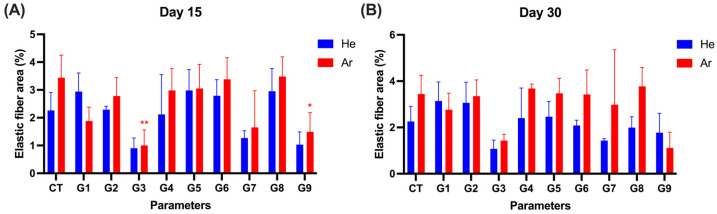

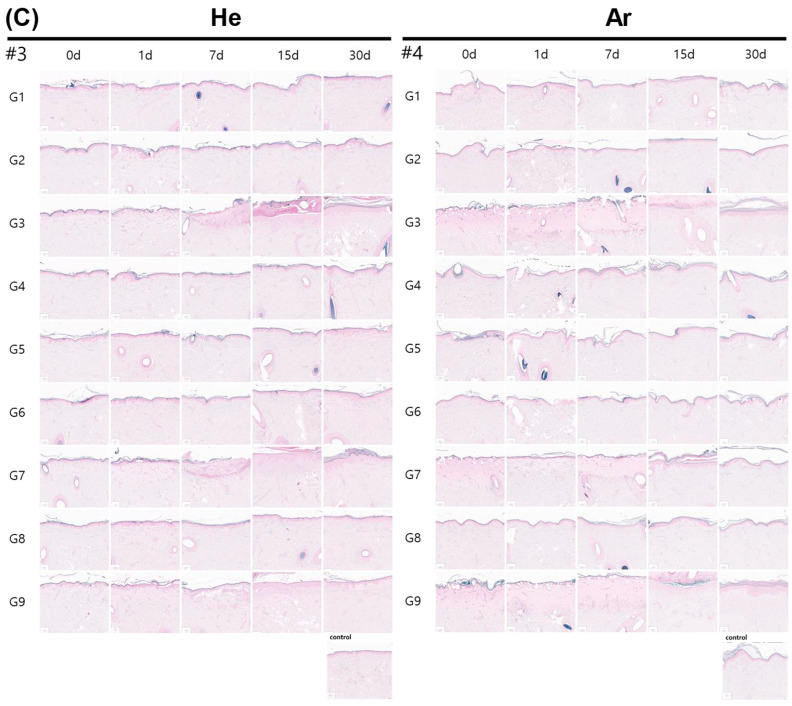
Changes in the proportion of elastic fibers at the application site. The area of elastic fiber was evaluated using Victoria blue staining on days 15 (**A**) and 30 (**B**). Victoria blue staining images (#3: Animal No. 3, #4: Animal No. 4) (**C**). Data was displayed as mean ± SD. G1–G9: 9 different parameter conditions of the plasma device; He: Helium gas resource, Ar: Argon gas resource; 0 d: 0 day (on the treatment day), 1 d: 1 day, 7 d: 7 days, 15 d: 15 days, 30 d: 30 days post-irradiation day. Significance levels were set at * *p* < 0.05, ** *p* < 0.01, data displayed as mean ± SD (*n* = 3). For comparison of the difference between the gas resources in each treatment condition, statistical analysis was assessed by two-way ANOVA analyses followed by a Sidak’s multiple comparisons test (asterisk in black). One-way ANOVA followed by Dunnett’s multiple comparisons test was performed to determine the difference between the control group and treatment conditions within each gas resource (asterisk in blue (Ar)/red (He)).

We assessed changes in dermal elastic fibers at the plasma application sites using Victoria blue (VB) staining. VB-stained tissues were observed and analyzed to measure the proportion of elastic fiber area on day 15 and 30 post-treatment (Figure 7 and Figure S5, and Table S7).

For the He gas conditions, no significant differences were observed in any treatment condition compared to the control group (2.26 ± 0.65%) (Figure 7A,C). Under conditions G3 and G9, a decrease in elastic fiber area was noted on day 15 and day 30, but there were not statistically significant relative to the control group.

For Ar gas conditions G1, G2, G4, G5, G6, G7, and G8, no significant differences were observed compared to the control group (2.26 ± 0.65%) (Figure 7A,C). However, under conditions G3 and G9, the elastic fiber area was significantly decreased on day 15 compared to the control group: G3 (*p* = 0.009) and G9 (*p* = 0.05) (Table S7). This reduction was not observed on day 30.

### 2.8. Evaluation of Elasticity Proliferation of Skin After Plasma Irradiation

**Figure 8:**
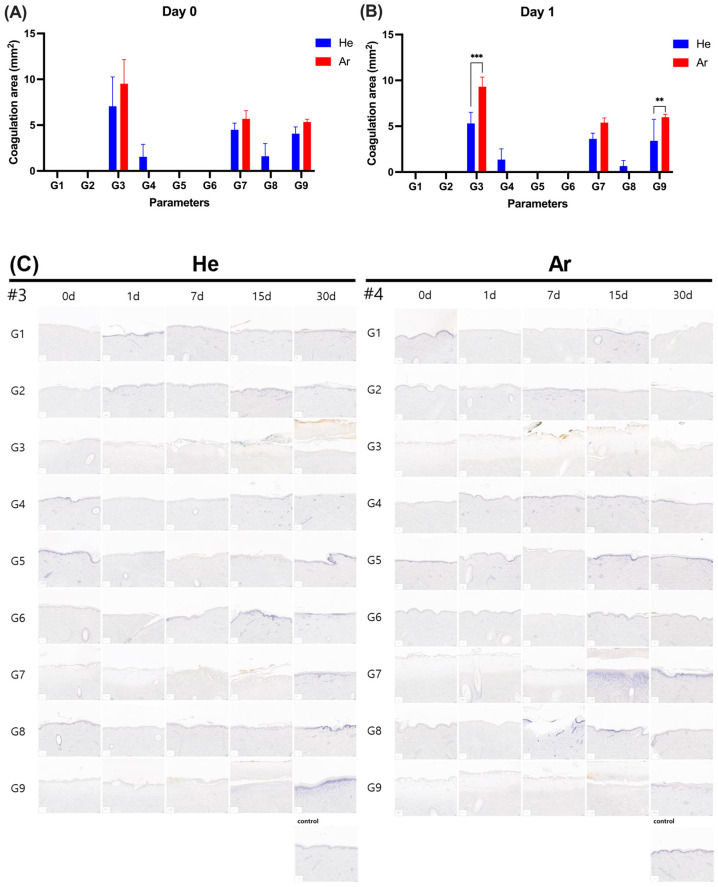
Elasticity proliferation of skin after irradiation. Histological sections with NBTC stain showed coagulation area (mm^2^) at the application site on day 0 (**A**) and day 1 (**B**), and NBTC staining images of skin tissue morphology for animal No. 3 and animal No. 4 (#3: Animal No. 3, #4: Animal No. 4) (**C**). G1–G9: 9 different parameter conditions of the plasma device; He: Helium gas resource, Ar: Argon gas resource; 0 d: 0 day (on the treatment day), 1 d: 1 day, 7 d: 7 days, 15 d: 15 days, 30 d: 30 days post-irradiation day. Significance levels were set at ** *p* < 0.01, *** *p* < 0.001, data displayed as mean ± SD (n = 3). For comparison of the difference between the gas resources in each treatment condition, statistical analysis was assessed by two-way ANOVA analyses followed by a Sidak’s multiple comparisons test (asterisk in black). One-way ANOVA followed by Dunnett’s multiple comparisons test was performed to determine the difference between the control group and treatment conditions within each gas resource (asterisk in blue (Ar)/red (He)).

To assess the coagulation area of tissues after plasma irradiation, tissue sections stained with the redox dye Nitro Blue Tetrazolium Chloride (NBTC) were examined at day 0 and day 1 post-irradiation, as shown in Figure 8 and Figure S6 and Table S8. No coagulation was observed under conditions G1, G2, G5, and G6 using He gas (Figure 8). In contrast, under conditions G3, G4, G7, G8, and G9 with He gas, coagulation areas were evident both immediately after irradiation (day 0) and on day 1 post-irradiation.

The coagulation was observed at 1.54 ± 1.37 mm^2^ and 1.35 ± 1.18 mm^2^ for G4; 4.49 ± 0.73 mm^2^ and 3.62 ± 0.62 mm^2^ for G7; 1.60 ± 1.39 mm^2^ and 0.66 ± 0.59 mm^2^ for G8; and 4.07 ± 0.73 mm^2^ and 3.40 ± 2.36 mm^2^ for G9 on day o and day 1, respectively (Table S8).

For Ar gas, no coagulation was observed under conditions G1, G2, G4, G5, G6, and G8. However, under conditions G3, G7, and G9, coagulation areas were observed: G3 (9.52 ± 2.64 mm^2^ immediately after irradiation, 9.30 ± 1.06 mm^2^ on day 1), G7 (5.68 ± 0.90 mm^2^ immediately after irradiation, 5.39 ± 0.52 mm^2^ on day 1), and G9 (5.35 ± 0.29 mm^2^ immediately after irradiation, 5.98 ± 0.32 mm^2^ on day 1) (Table S8). Notably, under treatment conditions G3 and G9, the Ar group showed a significant increase in coagulation area compared to He one day post-irradiation. These findings indicate that coagulation was condition-dependent and varied between the He and Ar gas-based plasma treatments.

## 3. Discussion

Plasma medicine is an emerging research domain with numerous potential applications across various fields, owing to its capacity to sterilize microorganisms, inhibit cancer cells, and stimulate tissue regeneration [31,32]. The design and adjustment of the conditions of cold atmospheric plasma (CAP) devices can be adjusted to achieve a variety of purposes. When optimized, CAP shows significant promise in wound management and treatment of dermatological conditions. Indeed, multiple randomized controlled trials report accelerated healing in venous leg ulcers and diabetic foot wounds treated with CAP compared to placebo or standard care, without any serious device-related adverse events [33,34,35]. Therefore, addressing the device’s safety and effectiveness is of utmost importance [36,37]. Hence, we conducted the current study to evaluate the effectiveness and safety of the PlazMagik device, a novel handheld cold atmospheric plasma generator. To assess the effectiveness of the device, we evaluated the impact of various treatment parameters, including the type of gas used (helium (He) and argon (Ar)), handpiece configuration (single or triple), operation mode (continuous or pulse), and output of setting of gas and plasma on a pig skin model (Table 1 summarizes these parameters). Following the device application, we monitored the clinical values of the experimental animal for 30 days, assessing body weight fluctuations, temperature at the application location, and blood biochemical characteristics, and we performed histopathological analysis.

Despite the fact that cold atmospheric plasmas are referred to as “cold,” they still emit thermal energy, with temperatures spanning from 30 °C to 100 °C. While temperatures exceeding 40 °C are not ideal for treating mammalian tissues, adjustments to the setting of the gas flow rate and the distance of the CAP resource from the target can help prevent excessive heating [8,38]. In terms of CAP devices used for skin regeneration operating with atmospheric air, the temperature can reach 60 °C in the dermis at a high energy setting [39,40].

In the current study, our treatment conditions showed that the skin surface temperatures did not exceed 60 °C. We observed a temperature rise on the skin surface due to plasma irradiation, regardless of the specific gas type, handpiece, or duration of exposure. This increase in skin temperature after plasma irradiation was consistent with previous studies that reported similar thermal responses during plasma treatments [8,41]. Although skin surface temperatures occasionally reached up to 57 °C, exposure duration was brief, and thus below the established thresholds for sustained tissue damage [42,43]. The mild epithelial cell changes seen under certain conditions (G3, G7, G9) likely reflect reversible thermal stress rather than irreversible burns. This is supported by full re-epithelialization and absence of full-thickness injury by day 15, consistent with recognized thermal dose responses.

When He gas was used as the plasma generator’s resource (He-CAP), a temperature rise was observed in two out of three animals under plasma output at 10, gas output at 5, and pulse mode (condition G4). Differences in the test start dates resulted in varied pulse irradiation times, with subjects 1 and 2 receiving 500 ms pulses compared to 100 ms for subjects 3 to 6, which likely caused the temperature increase in the former group. This incident indicates that an increase in the pulse duration of the plasma setting leads to a rise in temperature, which, as previously reported [44,45,46,47], results in damage to cells and tissues.

The variability observed in the pigs under G4 conditions (He-CAP) may stem from both technical and biological factors. As previously documented, pulse width profoundly influences CAP device effects and tissue interactions, with even small deviations significantly altering energy dose and biological outcomes; the optimal setting is typically in the microsecond range [48]. At the same time, physiological differences between animals—particularly those arising from genetic variation in glucocorticoid receptor (NR3C1) expression—have been shown to influence stress hormone (cortisol) responses with high heritability (h^2^ ≈ 0.64) [49,50]. Together, these technical and genetic factors may have contributed to differences in the histological responses between animals treated under nominally identical conditions. Therefore, future studies should ensure precise control of pulse duration, expand the sample size, and include physiological biomarkers (e.g., cortisol concentrations or GR genotype) to better explain and understand these sources of variability.

Compared with He gas (He-CAP), Ar gas (Ar-CAP) showed a temperature increase even under low-power conditions, using a single handpiece, plasma output set to 1, gas output set to 1, and continuous mode (condition G1). This result suggests that the output energy on the skin surface is greater with Ar gas, even during low-power irradiation modes such as G1. Previous studies have reported that this higher thermal effect of Ar may be attributed to the distinct physical properties of the two gases [51]. For instance, Aguilera & Aragón (1999) compared laser-produced plasma in air, argon, and helium, observing that the highest temperature and electron densities occurred in argon, while the lowest were found in helium [52]. Consistent with this finding, Yambe et al. (2024) demonstrated that the electron temperature of helium plasma is lower than that of argon plasma under similar conditions, attributing this difference to variations in collision frequency [53]. This distinction is particularly relevant for CAP devices used in biomedical research; the operational parameters for argon and helium are different. A comparison of CAP devices operated with argon or helium showed that the induced skin surface temperature is higher with argon than with helium [51]. Collectively, these findings align with our results, underscoring the critical importance of optimizing plasma operational parameters based on gas selection to minimize temperature increases on the skin while maintaining the device’s effectiveness.

We also evaluated the safety and the effects of He-CAP and Ar-CAP on changes in blood biochemical parameters. On the first day post-treatment, blood biochemical analysis revealed a slight increase in AST (aspartate aminotransferase), an enzyme found in various tissues such as skeletal muscle and livers [54]. This increase in AST level is likely due to temporary muscle damage or stress caused by the movement and anesthesia on the day of treatment [55,56]. However, no changes were observed in ALT or ALP levels, indicating that the treatments are safe and did not have a significant impact on major organ function [54,56].

We attribute the observed increase in AST to likely skeletal muscle injury related to animal movement and anesthesia. Although CK was not measured in this study, the literature indicates that CK and AST show strong concordance in muscle damage cases. The lack of a liver-specific enzyme (e.g., SDH, GLDH) and CK limits our ability to fully differentiate the AST’s source [57,58]. Future research should include simultaneous CK and AST measurements to definitively distinguish hepatic from muscular contributions.

Regarding changes in skin surface appearance, no adverse reactions were observed under low-energy output of plasma operational conditions, where neither gas caused a temperature increase. However, we observed significant differences on the skin’s surface when the high-energy output settings were applied, highlighting the distinct effects of each gas. With He-CAP, immediately after application, the skin surface in the exposed area turned white due to coagulation, followed by erythema and scabbing after seven days, reflecting the typical tissue healing response. Ar-CAP, on the other hand, caused direct skin damage, resulting in skin erosion and erythema. By day 7, scabbing had formed, similar to that seen with He-CAP, but with more severe damage, suggesting that Ar-CAP has a stronger effect on the skin, although the plasma jets were claimed to emit low-temperature plasma plumes in the surrounding air, suggesting they are safe for skin applications [59,60]. Due to their ability to sustain temperatures below 40 °C, they are capable of interacting with soft matter, such as biological tissues, without causing thermal injury [60,61]. Despite abundant previous research reporting the benefits of the CAP system in multiple applications [13,29,60], our current study revealed that high-energy-output operational parameters could cause skin surface damage. This finding aligns with previous studies reporting the risk of skin damage when using CAP-jet in both animal models and clinical trials [9,10,62,63]. Kos et al. previously demonstrated a correlation between prolonged treatment duration, increased gas flow rates, and the progression of skin damage. The authors indicated that both direct and indirect skin damage were correlated with increasing flow rates, elevated temperatures of treated skin, and the rise in RONS and streamer formation [62]. These findings highlight the necessity of meticulously choosing plasma operational parameters, including energy output and gas flow rate, to reduce negative skin reactions. Optimization and control of treatment conditions are essential for the safe and effective clinical application of cold atmospheric plasma, ensuring that therapeutic benefits are realized while maintaining tissue integrity.

Our histopathological analysis revealed that high-output groups, particularly with Ar-CAP, showed clear signs of coagulation, including basal and dermal layer separation, epithelial cell contraction, and vacuolization. While tissue regeneration was observed over 30 days, full recovery, especially in dermal thickness and the collagen fiber ratio, was not achieved. When comparing He-CAP and Ar-CAP at the same output levels, Ar-CAP caused a significantly larger coagulation area, indicating a more potent coagulation effect. The difference between He and Ar plasma jets is primarily due to their ionization energies. He metastable particles are more conducive to He plasma plume propagation, with He jets containing more O and N2+ whereas the Ar plasma jet contains more OH and N2. Ar has less ionization energy than He, so there are more ions and electrons in the Ar plasma jet discharge channel than in the He plasma jet. This means that there is a bigger discharge current. He metastable particles with a high energy and a long lifetime, on the other hand, are better for spreading the He plasma stream [64]. Moreover, argon-based cold atmospheric plasma (Ar-CAP) typically generates higher levels of hydroxyl radicals (·OH) compared to He-CAP and facilitates greater reactive nitrogen species (RNS) through enhanced N_2_ dissociation [65,66]. These reactive species synergistically oxidize proteins and lipids, induce nitrative cross-linking, and rapidly denature endothelial and extracellular matrix components, thereby promoting more extensive and faster coagulation compared to helium plasma [67].

We observed notable inter-animal variability, especially under helium plasma conditions like G4, where some pigs exhibited epithelial damage while others did not. This variability may reflect both genetic factors, such as glucocorticoid receptor polymorphisms and stress responses, and physiological differences like baseline cortisol levels, hydration status, or circadian variations [50,68]. Future studies should therefore expand cohort sizes and monitor genetic markers, cortisol dynamics, and standardized pre-treatment conditions to better understand and mitigate these sources of variability.

Several studies using porcine skin models have demonstrated the safety and tissue-compatible effects of cold atmospheric plasma (CAP). Dobrynin et al. (2011) showed that even CAP doses exceeding those used for microbial inactivation did not cause tissue damage in live pig skin, while enhancing coagulation [69]. Similarly, Maisch et al. (2012) reported no histological harm following CAP treatment of ex vivo pig skin [70]. These results align with ours, where certain plasma conditions induced only transient epithelial changes followed by full re-epithelialization. Moreover, a recent review highlights that, although prolonged or high-intensity exposure can impair pig skin, shorter, optimized CAP treatments consistently preserve tissue integrity during wound healing [71]. By directly comparing helium and argon plasmas over a 30-day period in live animals, our study adds a new dimension, revealing gas- and condition-specific effects on dermal morphology, healing kinetics, and extracellular matrix remodeling. This extends existing knowledge by showing that both He-CAP and Ar-CAP can be safe and effective when used within appropriately defined exposure parameters.

While our study provides valuable insights into the importance of the operation conditions of the cold plasma device and its gas resource, it is important to acknowledge that there are some limitations due to the lack of comprehensive research in this area. Firstly, this study contained a limited number of experimental animals, which should incorporate larger sample sizes to ensure more robust and reliable results in future research. The observation duration of 30 days may not be sufficient to capture long-term effects comprehensively. Furthermore, additional research on uncompromised skin conditions could provide more evidence for optimizing the settings of this device for skin therapy applications.

Our study focused on visual, biochemical, and histological endpoints; future work should integrate functional skin assessments—such as elasticity via ultrasound or optical elastography, hydration metrics, and molecular biomarkers of aging or inflammation (e.g., MMPs, cytokines). These non-invasive measurements have shown promise in porcine and human skin models for capturing biomechanical and structural changes. Including such metrics in longer-term, larger-scale studies would provide valuable mechanistic insight and strengthen the translational relevance of PlazMagik treatments.

Despite these limitations, our study offers preliminary evidence highlighting the importance of the mechanical setup and plasma gas sources for the performance of the helium/argon plasma jet device used in this study.

## 4. Materials and Methods

### 4.1. Ethical Approval

This study was conducted in compliance with the animal testing ethics regulations of the Osong Advanced Medical Industry Promotion Foundation, a public institution under the Ministry of Health and Welfare of the Republic of Korea that has been certified by AAALAC International (The Association for Assessment and Accreditation of Laboratory Animal Care) and KELAF (Korean Excellent Animal Testing Facility) (IACUC, No. KBIO-IACUC-2023-161).

### 4.2. Plasma Equipment

The helium/argon plasma jet device was developed by AGNES Medical Co., Ltd (Seongnam-si, Republic of Korea). and contains optional nozzle types designed for specific treatment goals (single or triple nozzle handpieces). The device is designed to operate with alternating current (AC) and can accept a range of input voltages from 100 to 240 volts and frequencies of 50 or 60 Hz. The plasma vehicle gas, argon (99.999%) or helium (99.999%) gas, was used with the same gas flow rate of 0.12 L/min to generate physical plasma using a 24 kHz frequency with 1000 W power (peak voltage of 6 kV).

### 4.3. Animals, Breeding Conditions, and Experiment Handling

This study was conducted on a total of 6 healthy female pigs (Yorkshire×Berkshire×Duroc (YBD crossbreeds)) at 4 months old, and weighing around 40 kg, acquired from Cronex Co., Ltd. (Hwaseong-si, Republic of Korea).

When acquiring the animals, inspection and quarantine were conducted by referring to the health monitoring report provided by the supplier. General symptoms were observed during the 7-day acclimatization period to confirm that they were healthy before being used in the experiment.

#### 4.3.1. Environmental Conditions

This experiment was conducted at Osong High-tech with a temperature of 23 ± 2 °C, relative humidity of 50 ± 10%, ventilation frequency of 10 to 15 times/hr, lighting time of 12 h (lights on from 8 a.m. to 8 p.m.), and illuminance of 150 to 300 Lux. The study was conducted in the pig rearing room and operating room within the Medical Industry Promotion Foundation’s Non-Clinical Support Center. In environmental monitoring, temperature, relative humidity, ventilation frequency, and illumination intensity were well maintained without deviation. All experimenters conducted the experiment wearing high-pressure steam-sterilized (121 °C, 20 min) work clothes and protective gear.

#### 4.3.2. Identification of Cage, Breeding Density, and Breeding Box

The animals were raised at 1 animal in a cage. To improve animal welfare, a ball with uneven surfaces was provided as environmental enrichment.

#### 4.3.3. Method of Feeding, Feed, and Water

The feed supplied for the animal was solid feed for the test animals (pig chow, Cargill & Grippurina B) sterilized by irradiation. The water was sterilized through RO water (reverse osmosis distilled water) and a UV device, and then freely consumed through an automatic water nozzle. The water was requested from an authorized institution (Chungbuk Institute of Health and Environment, Cheongju-si, Republic of Korea). As a result of regular water quality tests, it was judged to comply with drinking water quality standards.

#### 4.3.4. Experiment Design

The whole of the animal experiment setup complied with the ARRIVE guidelines. The animals were randomly divided and labeled from No. 1 to No. 6 and treated with 9 different parameter conditions of the plasma device, as presented in Table 1. The random number labels were generated using the standard = RAND () function in Microsoft Excel. While animals No. 1, No. 2, and No. 3 were irradiated with the He gas resource (*n* = 3), animals No. 4, No. 5, and No. 6 were treated with the Ar gas resource (*n* = 3) at the same setting conditions and labeled as in Table 1. Each treatment condition of the device was applied evenly spaced, with an interwound distance of 4–5 cm. The treatment was on pre-marked skin sites of approximately 3–4 cm^2^. The treatments were conducted under the same room conditions of 23 ± 2 °C and relative humidity of 50 ± 10%. Three different investigators participated in the investigation of each animal. The first investigator consistently treated the animals with the indicated conditions and gas resources and was the only person aware of the treatment condition allocation. The second investigator was involved in the anesthetic and performed the tissue sample collection. Lastly, a third investigator who was unaware of the treatment performed analyzed the encoded sample’s measurements.

#### 4.3.5. Application of Anesthesia and Testing Equipment

After injection of anesthesia with Zoletil^®^ 50 (Virbac Korea, Republic of Korea) at 5 mg/kg and Rompun^®^ (Bayer Korea, Seoul, Republic of Korea) at 2 mg/kg, an inhalation anesthetic machine (Fabius GS premium, Drager Medical, Lubeck, Germany) was used to maintain isoflurane (1.5–2%) using a mask for inhalation anesthesia. Afterward, we treated the indicated animals’ dorsal skin under the conditions listed in groups G1 to G9 in Table 1.

### 4.4. Skin Surface Temperature Evaluation

To observe changes in skin temperature immediately after plasma irradiation using PlazMagik equipment, skin surface temperature was measured by taking pictures with a thermal camera FLIR C5 (Teledyne FLIR LLC, Wilsonville, OR, USA).

### 4.5. Skin Visual Observation

To check the side effects on the skin surface caused by plasma irradiation, the skin condition was confirmed by imaging of the irradiated area with a dermoscope (IDS-1100, Illuco, Gunpo-si, Republic of Korea) at the indicated experimental time points.

### 4.6. Blood Biochemical Evaluation

Tests were conducted to observe hematological changes resulting from the plasma irradiation. Blood collected from the ear vein was placed in a serum separation tube (SST tubes) (BD Biosciences, Franklin Lakes, NJ, USA) and centrifuged at 2500 rpm for 15 min to separate serum. BUN, creatinine, calcium, and inorganic phosphorus were analyzed using a konelab PRIME 60i machine (Thermo Scientific, Waltham, MA, USA). Blood serum protein, albumin, A/G ratio, total bilirubin, AST, ALT, and ALP were also measured.

### 4.7. Histopathological Evaluation

On the final testing day (day 30), the animals were administered Zoletil^®^ 50 (5 mg/kg) and Rompun^®^ (2 mg/kg) intramuscularly for anesthetizing, followed by an intravenous injection of KCl for euthanasia. All the animals included in the study completed the entire experimental protocol; no subjects were excluded. The application area (skin on the back of the pig) was collected and investigated under the conditions of groups G1 to G9.

For histopathological evaluation of the skin after irradiation, tissue from the irradiated area was collected using an 8 mm biopsy punch immediately after irradiation or at 1, 7, 15, and 30 days after irradiation. All the samples were consistently taken from the center of each pre-marked treatment site to standardize sampling across all the conditions.

For comparable evaluation of the effect of each irradiation condition on the animal skin, normal skin at the corresponding area was collected at the same indicated time points and used as the control non-treatment samples. The collected tissue was divided into two halves; one was fixed with a 10% formalin solution, and a paraffin block was created through dehydration, transparency, infiltration, and embedding, followed by H&E, Masson’s trichrome (MT), and Victoria blue (VB) staining. The remaining tissues were quickly frozen using liquid nitrogen and stored in a deep freezer, and then NBTC staining was performed. Tissue slides were photographed with a slide scanner (panoramic scan, 3D histech, Budapest, Hungary). To evaluate the epithelial thickness, nine measurements (*n* = 9) were performed on three different area of the H&E staining sample. Three different measurements (*n* = 3) were performed for each treatment conditions to determine the collagen deposition rate (%), elastic fiber area (%), and coagulation area (mm^2^) using MT, VB, and NBTC staining samples.

### 4.8. Evaluation of the Coagulation Area

The width (a) and depth (b) of the tissue coagulation area were measured, and the area of the coagulation area (A) was calculated according to the following ellipse area formula.

$$Coagulation\;area\left(A\right)=\frac{a}{2}*\frac{b}{2}*\pi$$

### 4.9. Statistical Analysis

The results were presented as mean ± SD and the significant differences between two gas resources within each treatment condition were determined using two-way ANOVA along with Sidak’s multiple comparisons test.

The significant differences between the treatment group and normal group were determined using one-way ANOVA follow by Dunnett’s multiple comparisons test.

We analyzed the data and generated graphs using GraphPad Prism 9 (San Diego, CA, USA). Significance levels were set at * *p* < 0.05, ** *p* < 0.01, and *** *p* < 0.001.

## 5. Conclusions

In conclusion, our study showed that both He-CAP and Ar-CAP are safe when used appropriately at the lowest output plasma and gas mode under pulse operation modes. Meanwhile, under the same operating conditions, Ar-CAP’s stronger coagulation effects make it suitable for applications requiring more intense tissue alteration, while He-CAP may be better for milder treatments.

Our 30-day porcine study indicates that PlazMagik treatments under conditions G2, G4, G5, G6, and G8—across both helium and argon plasma modes—demonstrate the most favorable safety profile. These settings, representing the lowest energy and pulse parameters, induced no observable histological damage, maintained normal elastic fiber integrity, and showed only mild, reversible epithelial remodeling. These features make them particularly well-suited to mild skin rejuvenation applications that require minimal tissue disruption.

Our study highlights the importance of optimizing plasma parameters to maintain device effectiveness and warrants further investigation into the long-term effects with a larger number of animals and the recovery processes for both gases. Additionally, our data provides valuable insights into the device’s setup for addressing aesthetic concerns, particularly in managing skin regeneration and scar formation for future clinical applications.

## Figures and Tables

**Table 1:** The parameters and clinical outcomes of each setting condition.

Condition Name	Parameter Conditions					
	Handpiece Type	Plasma Output * (kV)	Gas Output Mode (kPa)	Operation Mode *	Irradiation Time (s)	Clinical Outcomes
G1	Single ^#^	2.6 ± 10%	7 ± 20%	Continuous	10 s	Safe but Ar gas showed immediately epithelial cell shrinkage
G2	Single	2.6 ± 10%	7 ± 20%	Pulse	10 s	Gentle and safe
G3	Single	4 ± 10%	11 ± 20%	Continuous	10 s	Skin erosion and erythema
G4	Single	4 ± 10%	11 ± 20%	Pulse	10 s	Gentle and safe
G5	Triple ^##^	2 ± 10%	13.5 ± 20%	Continuous	10 s	Gentle and safe
G6	Triple	2 ± 10%	13.5 ± 20%	Pulse	10 s	Gentle and safe
G7	Triple	3.2 ± 10%	21.5 ± 20%	Continuous	10 s	Skin erosion and erythema
G8	Triple	3.2 ± 10%	21.5 ± 20%	Pulse	10 s	Gentle and safe
G9	Single	4 ± 10%	11 ± 20%	continuous	5 s	Skin erosion and erythema

^#^ Single tip; diameter: Ø 22.5 mm, length: 27 mm; ^##^ Triple tip; diameter: Ø 41.7 mm, length: 34 mm; * Pulse mode: 3 Hz/166 ms.

## Data Availability

The data presented in this study is available on request from the corresponding author.

## Supplementary Materials

The following supporting information can be downloaded at: https://www.mdpi.com/article/10.3390/ijms26167854/s1.

## Abbreviations

The following abbreviations are used in this manuscript:

ALB	Albumin
ALP	Akaline phosphatase
ALT	Alanine aminotransferase
Ar	Argon
ARRIVE	Animal Research: Reporting of In Vivo Experiments
AST	Aspartate transaminase
BUN	Blood urea nitrogen
Ca	Calcium
CAP	Cold atmospheric plasma
Cr	Creatinine
H&E	Hematoxylin and eosin
He	Helium
IP	Inorganic phosphorus
MT	Masson’s trichrome
NBTC	Nitro Blue Tetrazolium Chloride
T-bil	Total bilirubin
TP	Total protein
UV	Ultraviolet
VB	Victoria blue
