# Supplementary material for: Assessment of a Helium/Argon-Generated Cold Atmospheric Plasma Device’s Safety Utilizing a Pig Model
Source: Int J Mol Sci. 2025 Aug 14;26(16):7854. doi: 10.3390/ijms26167854 (PMC12386185; doi:10.3390/ijms26167854)
Supplement: Supplementary file 1 [file ijms-26-07854-s001.zip › ijms-3749988-supplementary.pdf]

Title:

Preclinical research on optimization and validation of the safety and effectiveness of a helium/argon generated plasma device using pig model

Xin Rui Zhang [1,2,3#], Thuy-Tien Thi Trinh [1,4#], Linh Le Thi Thuy [1,5,6], Nguyen Ngan Giang [1,7], Yong Xun Jin [1,2], Young-Hyun Lee [4], Gun Young Ahn [8], Boncheol Leo Goo [9], Kyoung Su Jung [9], Hyun Soo Hwang [10], Pham Ngoc Chien [1,4,*], and Chan-Yeong Heo [1,2,4,7,*]

Supplementary Data:
Figures:
Figure S1. Body weight change of animals during 30 days of the experiment period.
Figure S2. Observation of skin change using dermatoscope after plasma irradiation.
Figure S3. H&E staining images.
Figure S4. Masson's trichrome staining images.
Figure S5. Victoria blue staining images.
Figure S6. NBTC staining images.

Tables:
Table S1. Observation of animal body weight during the experiment period.
Table S2. The temperature of the skin surface immediately after the irradiation.
Table S3. Blood serum chemistry measurement at pre, 0, 1, 7, 15, 30 days of He gas condition.
Table S4. Blood serum chemistry measurement at pre, 0, 1, 7, 15, 30 days of Ar gas condition.
Table S5. Thickness of the epithelial tissue in the application site (μm).
Table S6. Collagen deposition rate at the application site (%). Collagen density was evaluated using maason's trichrome staining.
Table S7. Changes in the proportion of elastic fibers at the application site (%). Area of elastic fiber was evaluated using Victoria blue staining.
Table S8. Plasma irradiation induced coagulation area (mm2). The coagulation area was evaluated using NBTC staining.

FIGURES

Figure S1. Body weight change of animals during 30 days of the experiment period. 1-6: labeled number of animals. Animal #1 to N#3: Animals were treated with CAP using Helium gas resource. Animal #4 to N#6: Animals were treated with CAP using Argon gas resource.

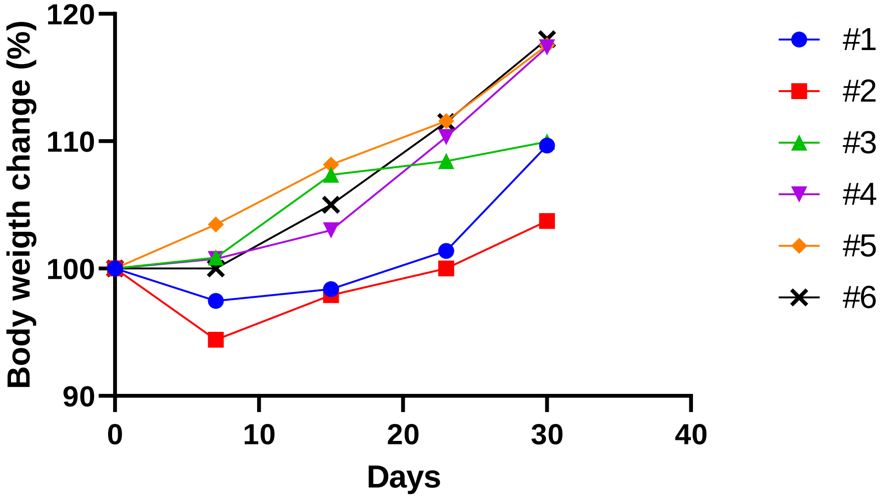

Figure S2. Observation of skin change using dermatoscope after plasma irradiation.

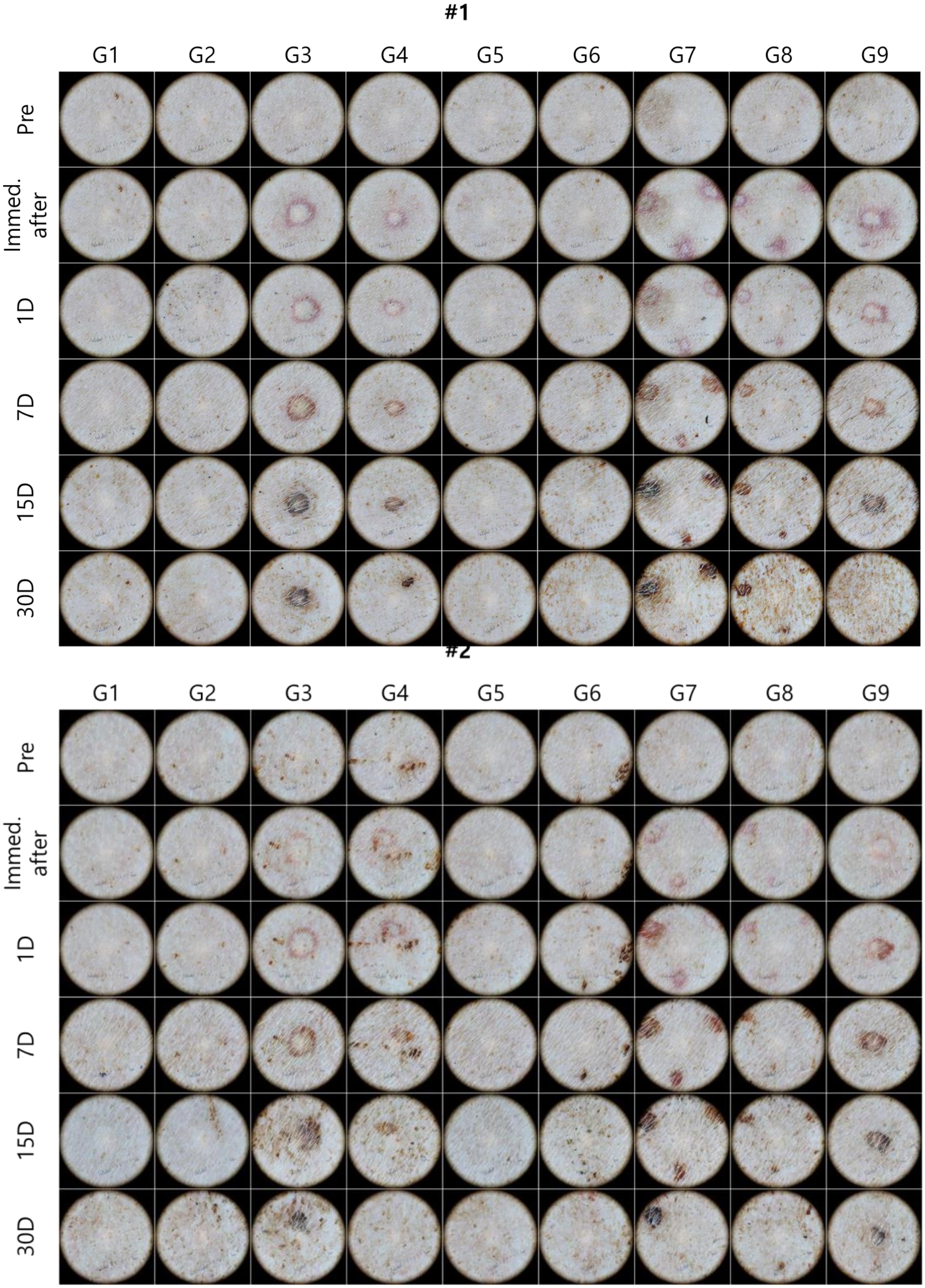

#3

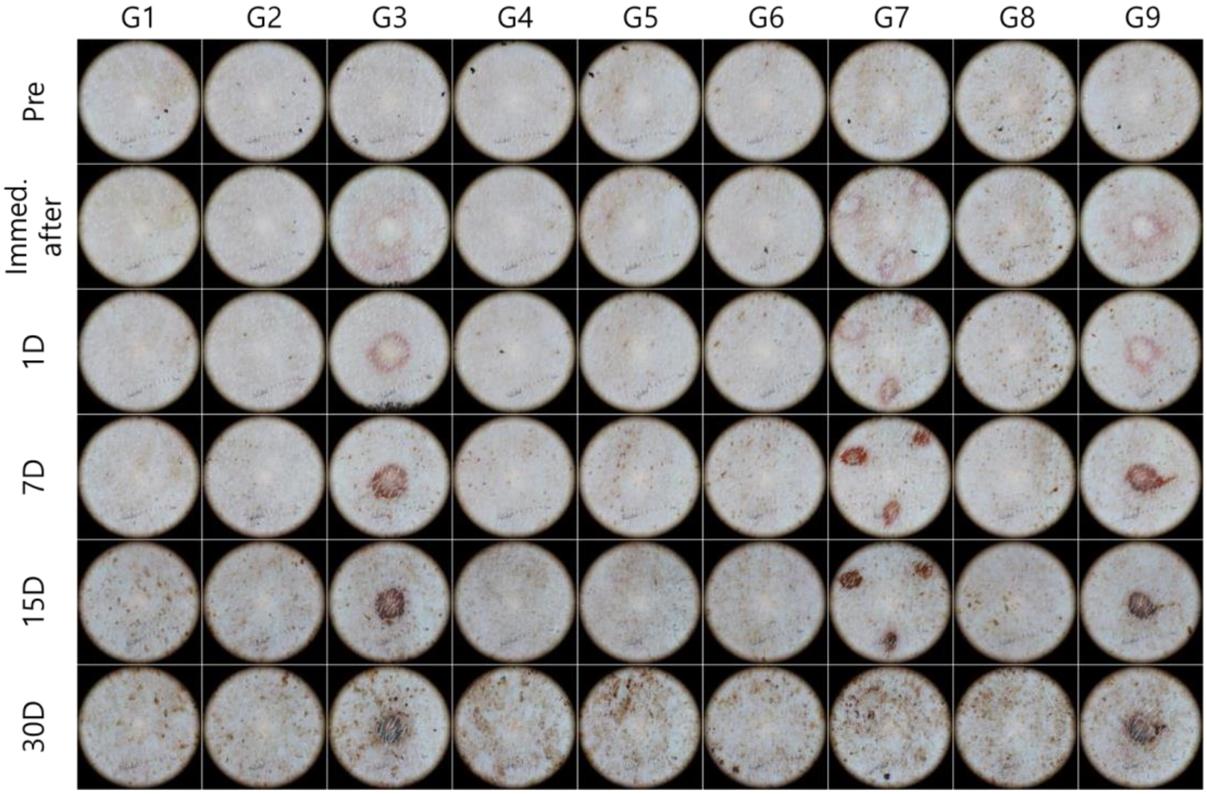
G1
G2
G3
G4
G5
G6
G7
G8
G9
Pre
Immed. after
1D
7D
15D
30D

#4

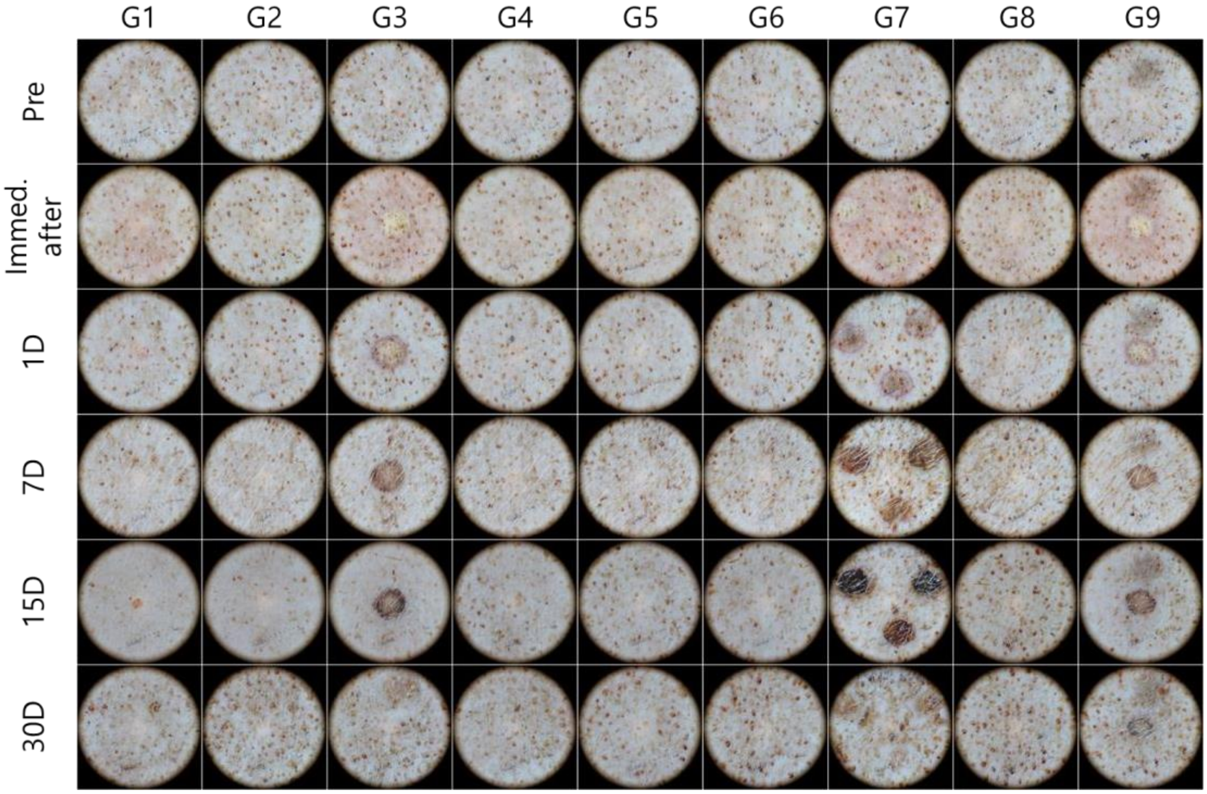
G1
G2
G3
G4
G5
G6
G7
G8
G9
Pre
Immed. after
1D
7D
15D
30D

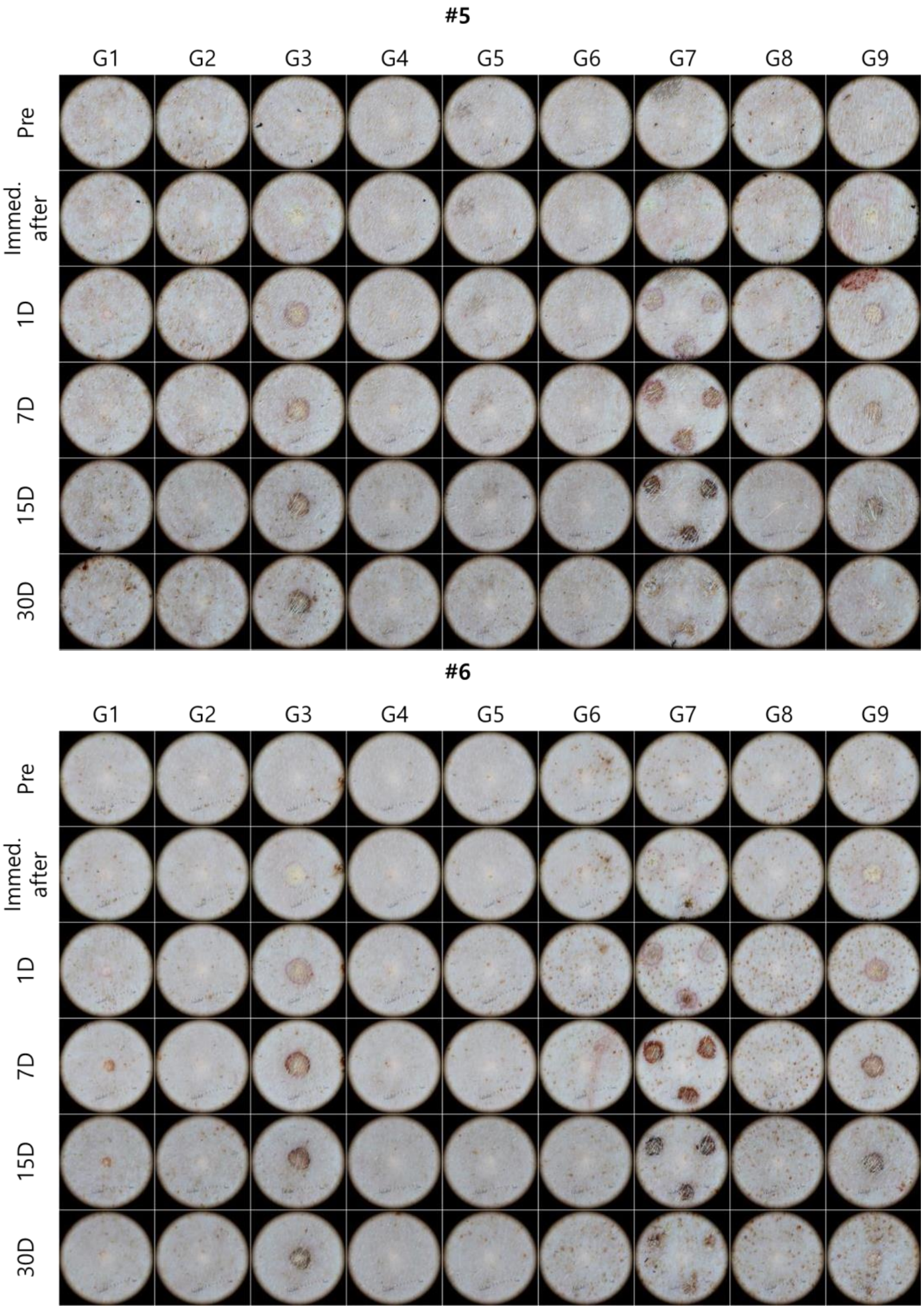
#5
G1
G2
G3
G4
G5
G6
G7
G8
G9
Pre
Immed. after
1D
7D
15D
30D
#6
G1
G2
G3
G4
G5
G6
G7
G8
G9
Pre
Immed. after
1D
7D
15D
30D

Figure S3. H&E staining images.

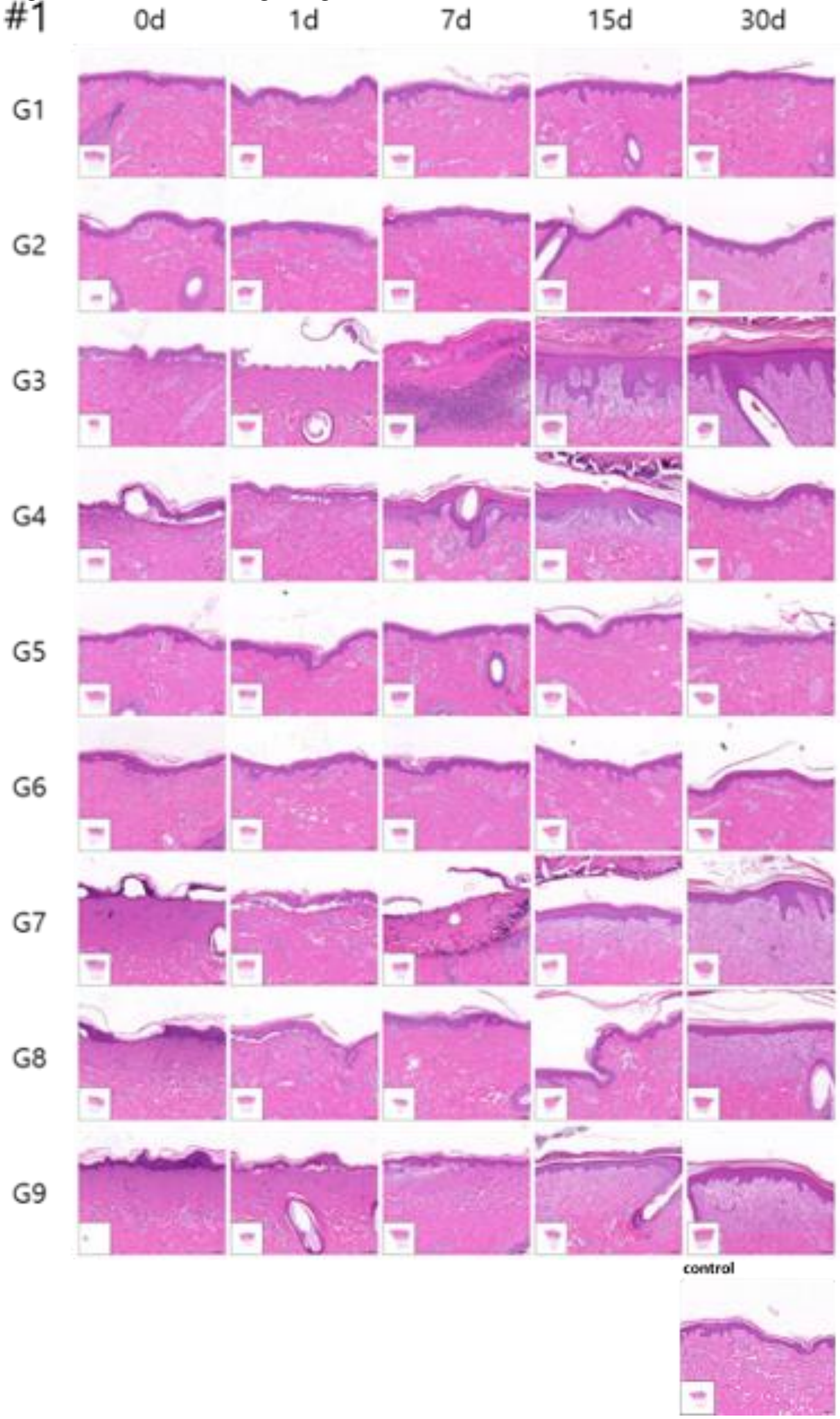

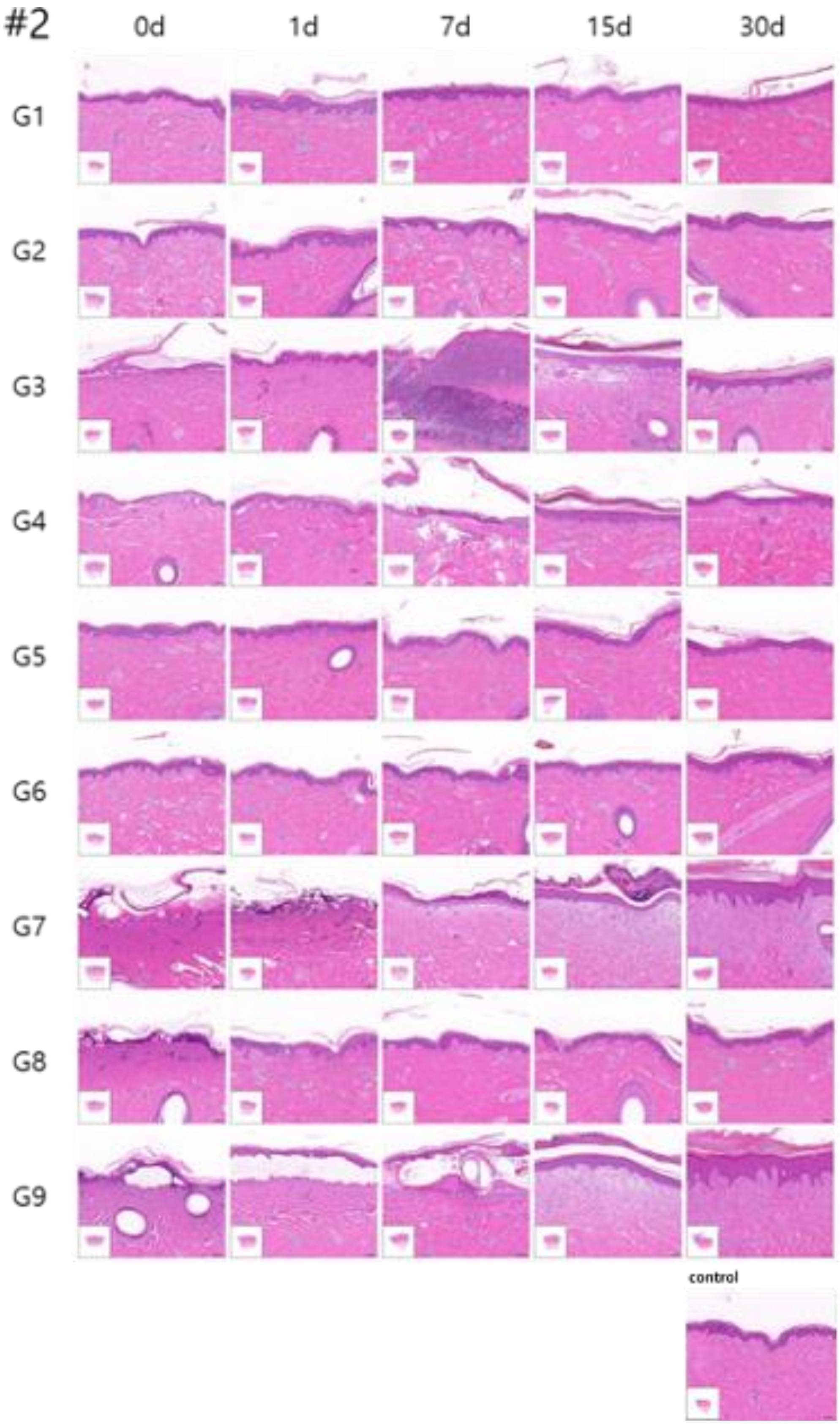
#2
0d
1d
7d
15d
30d
G1
G2
G3
G4
G5
G6
G7
G8
G9
control

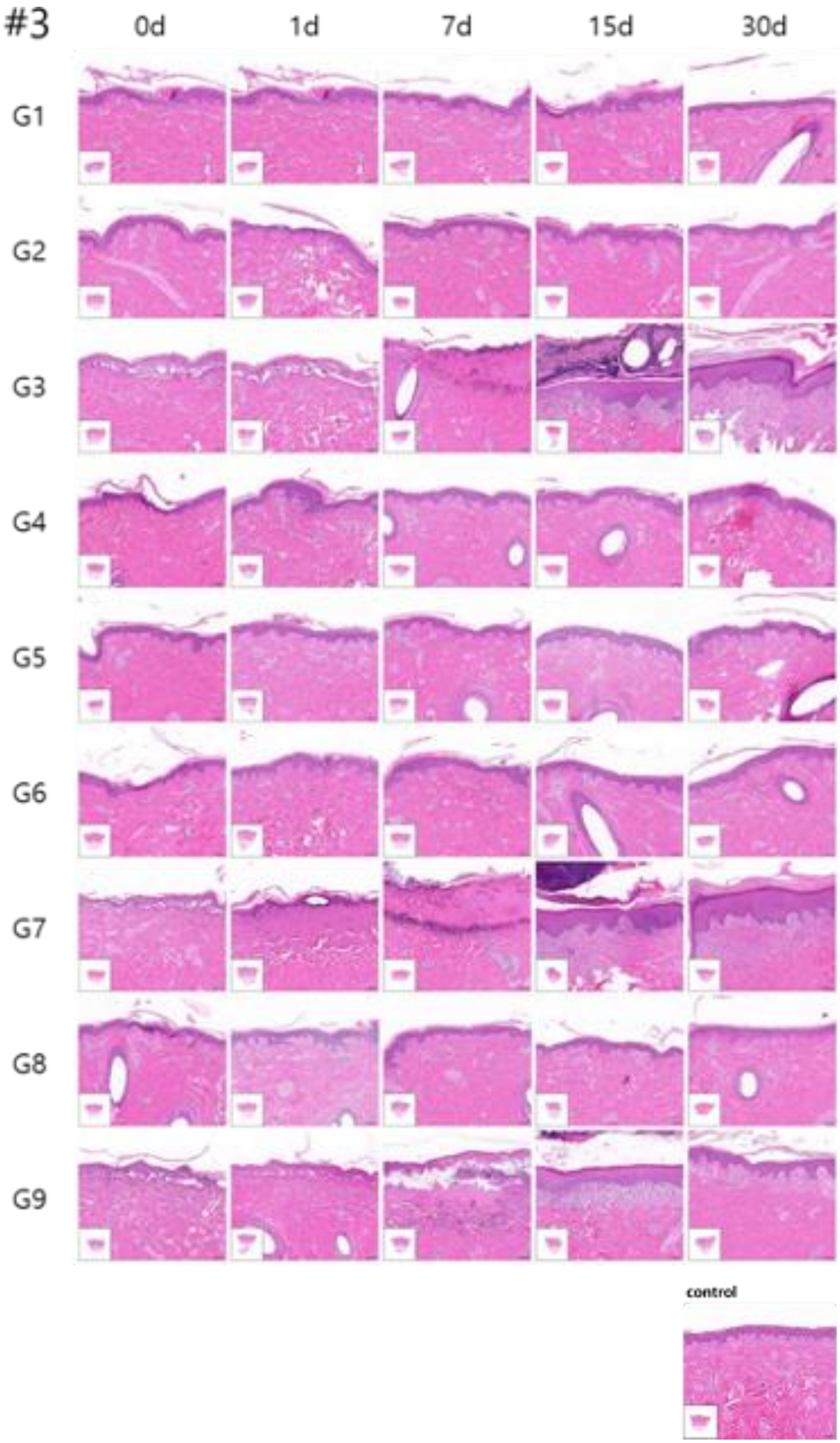
#3
0d
1d
7d
15d
30d
G1
G2
G3
G4
G5
G6
G7
G8
G9
control

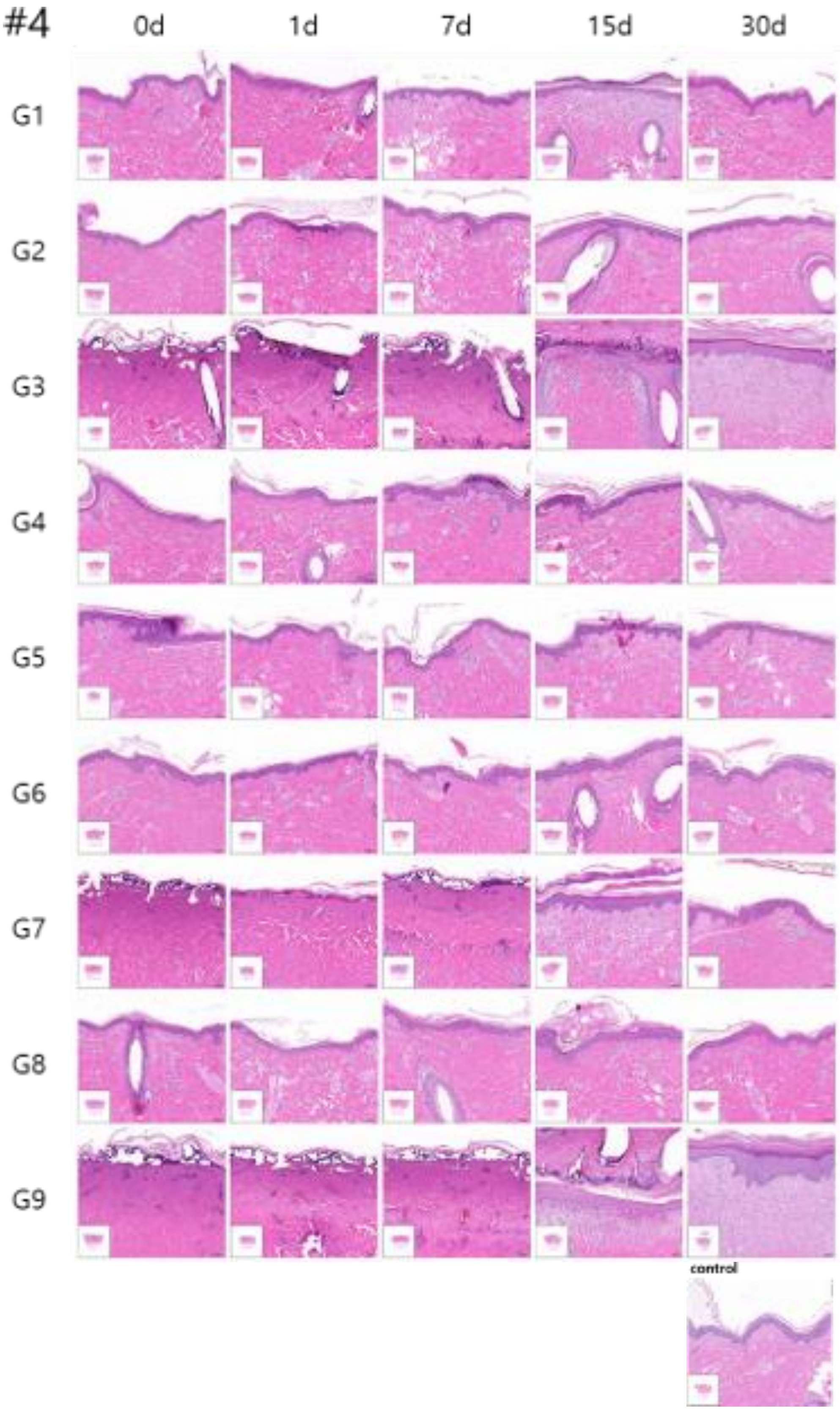
#4
0d
1d
7d
15d
30d
G1
G2
G3
G4
G5
G6
G7
G8
G9
control

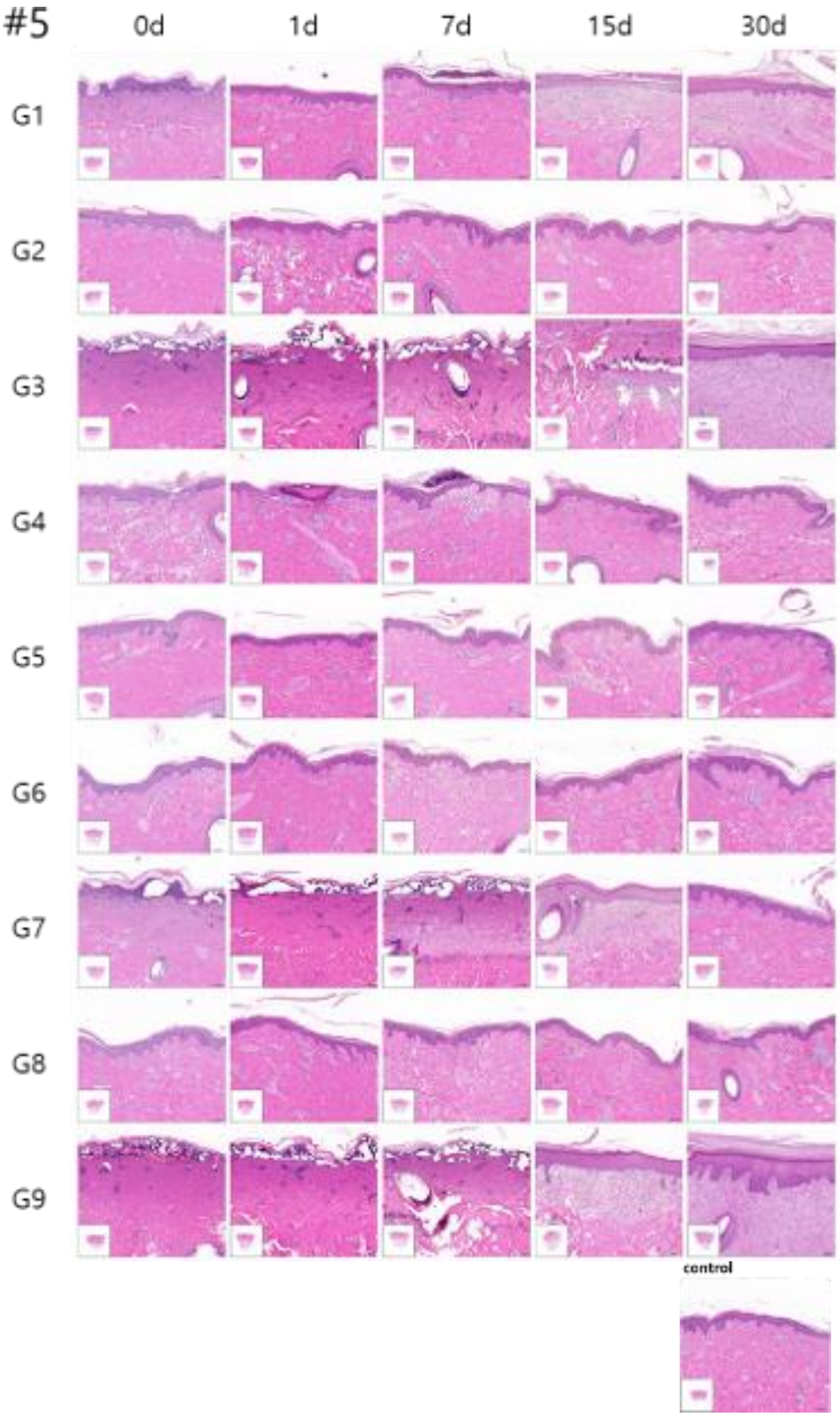
#5
0d
1d
7d
15d
30d
G1
G2
G3
G4
G5
G6
G7
G8
G9
control

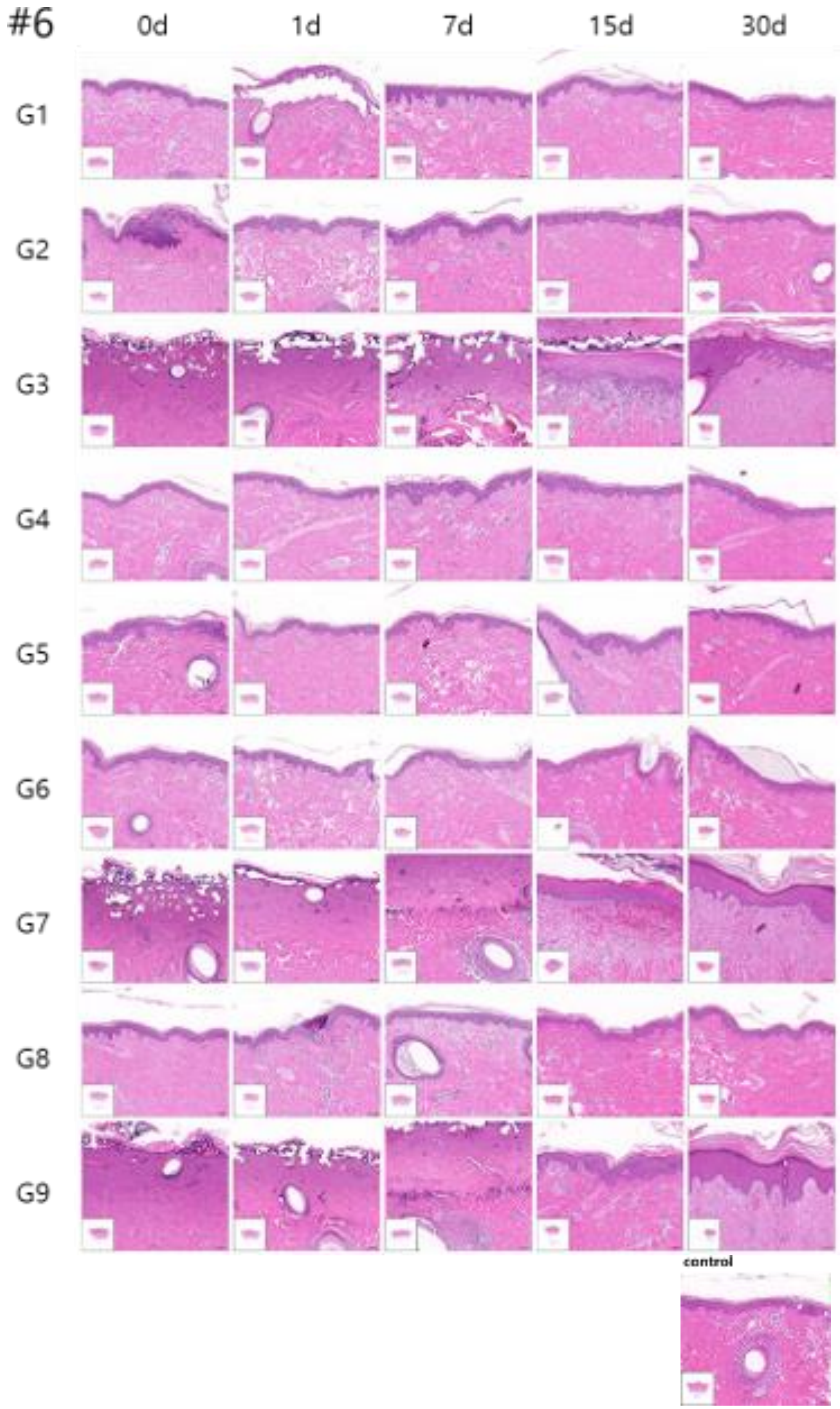

Figure S4. Masson's trichrome staining images.

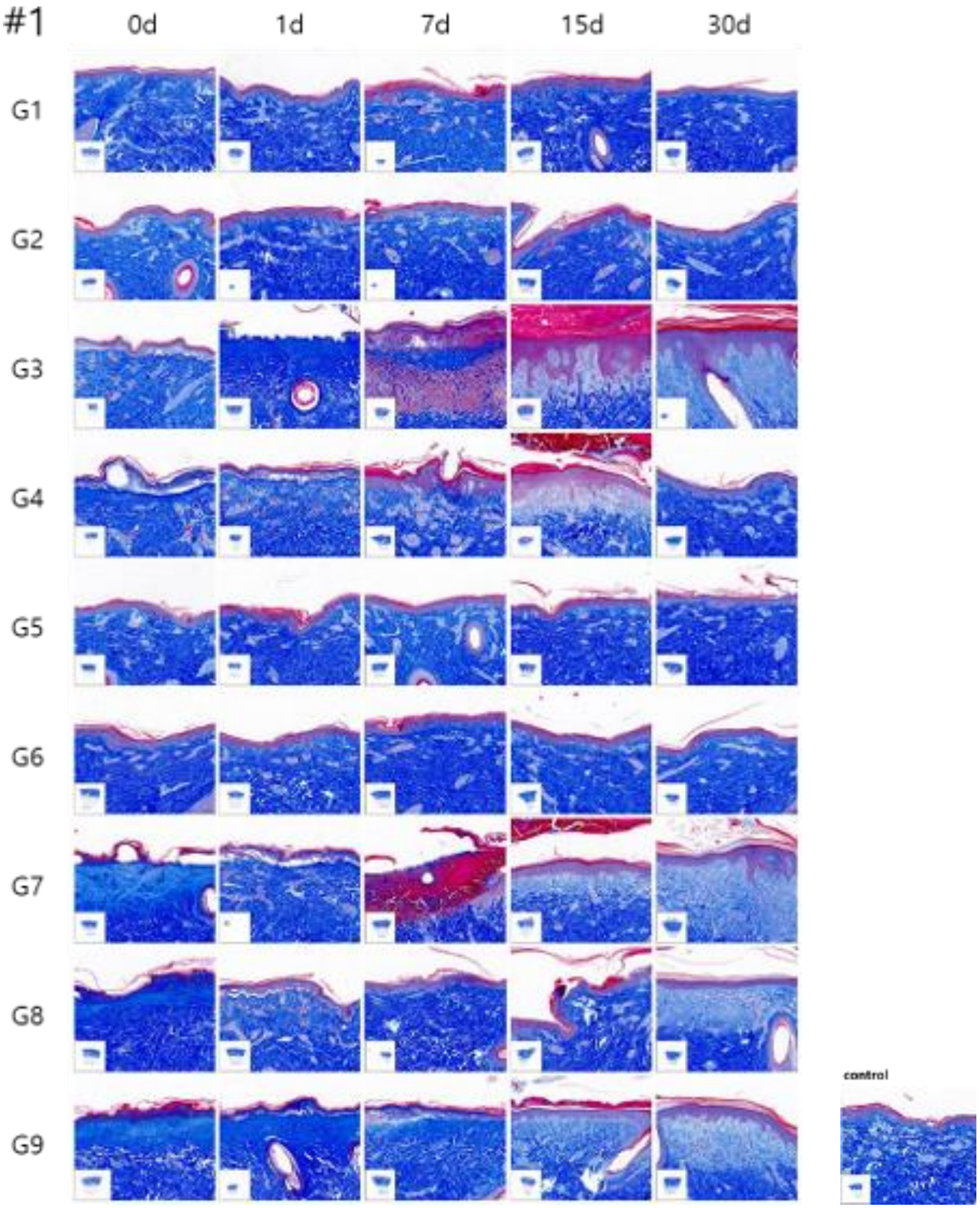
#1
0d
1d
7d
15d
30d
G1
G2
G3
G4
G5
G6
G7
G8
G9
control

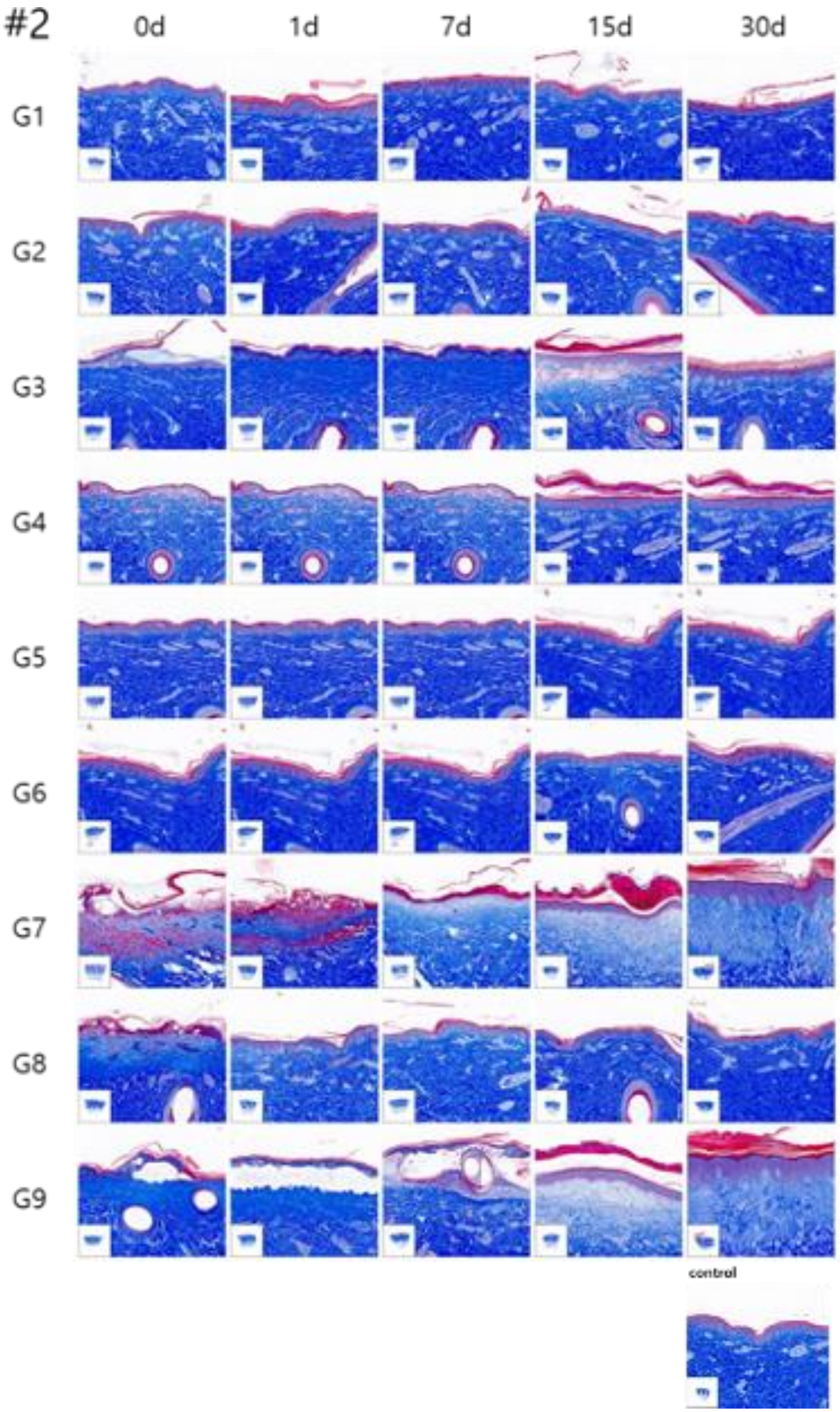

#2
0d
1d
7d
15d
30d
G1
G2
G3
G4
G5
G6
G7
G8
G9
control

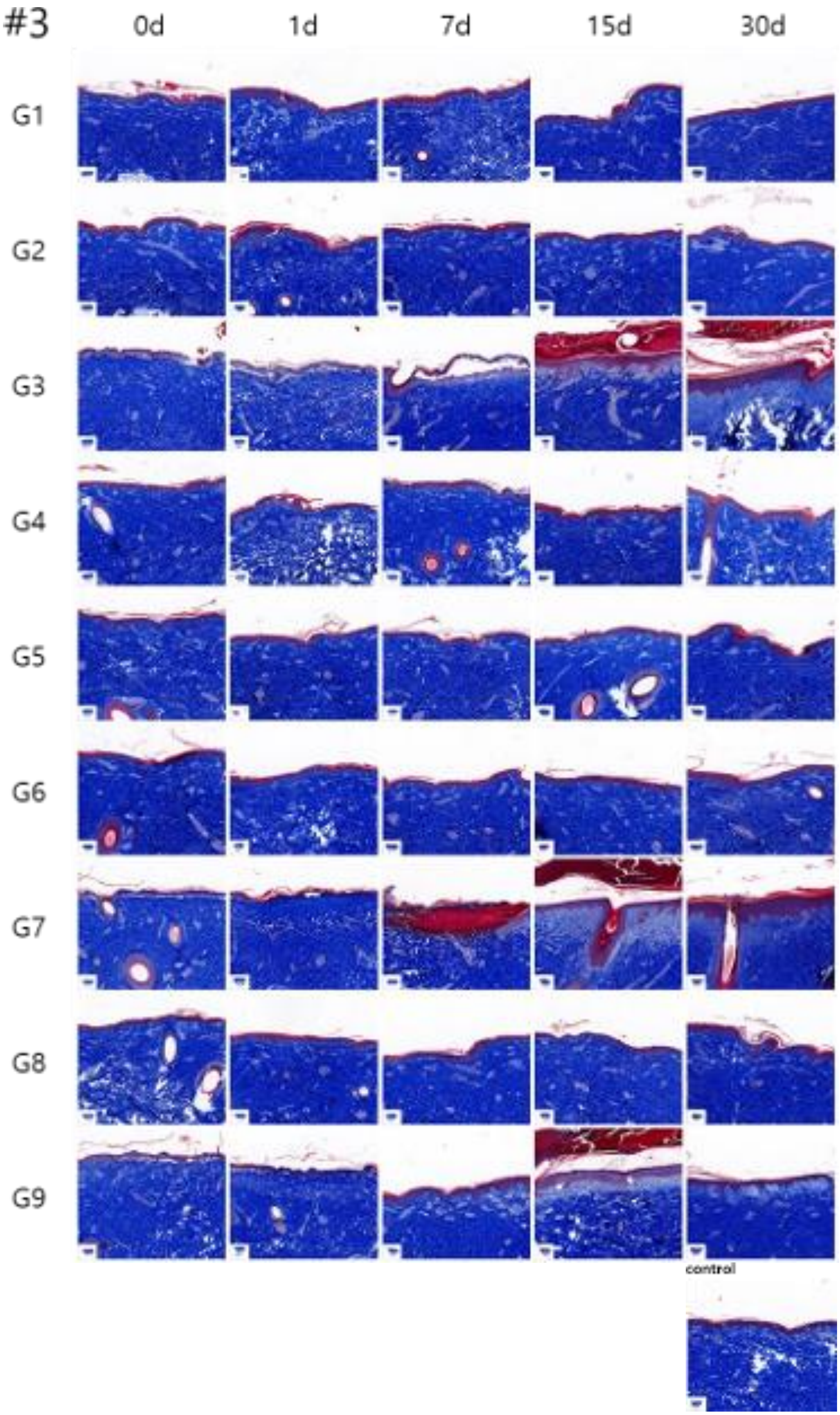

#3
0d
1d
7d
15d
30d
G1
G2
G3
G4
G5
G6
G7
G8
G9
control

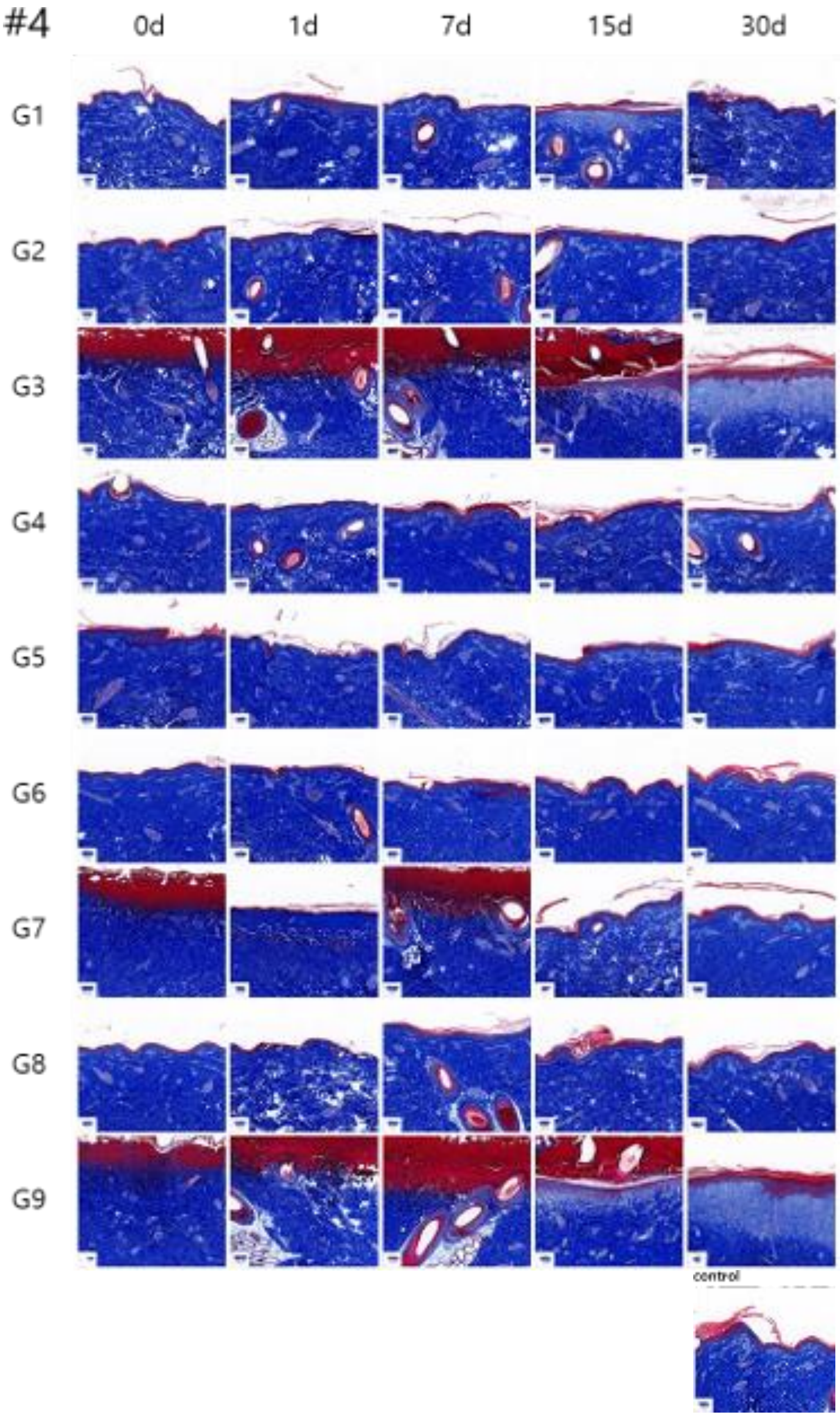
#4
0d
1d
7d
15d
30d
G1
G2
G3
G4
G5
G6
G7
G8
G9
control

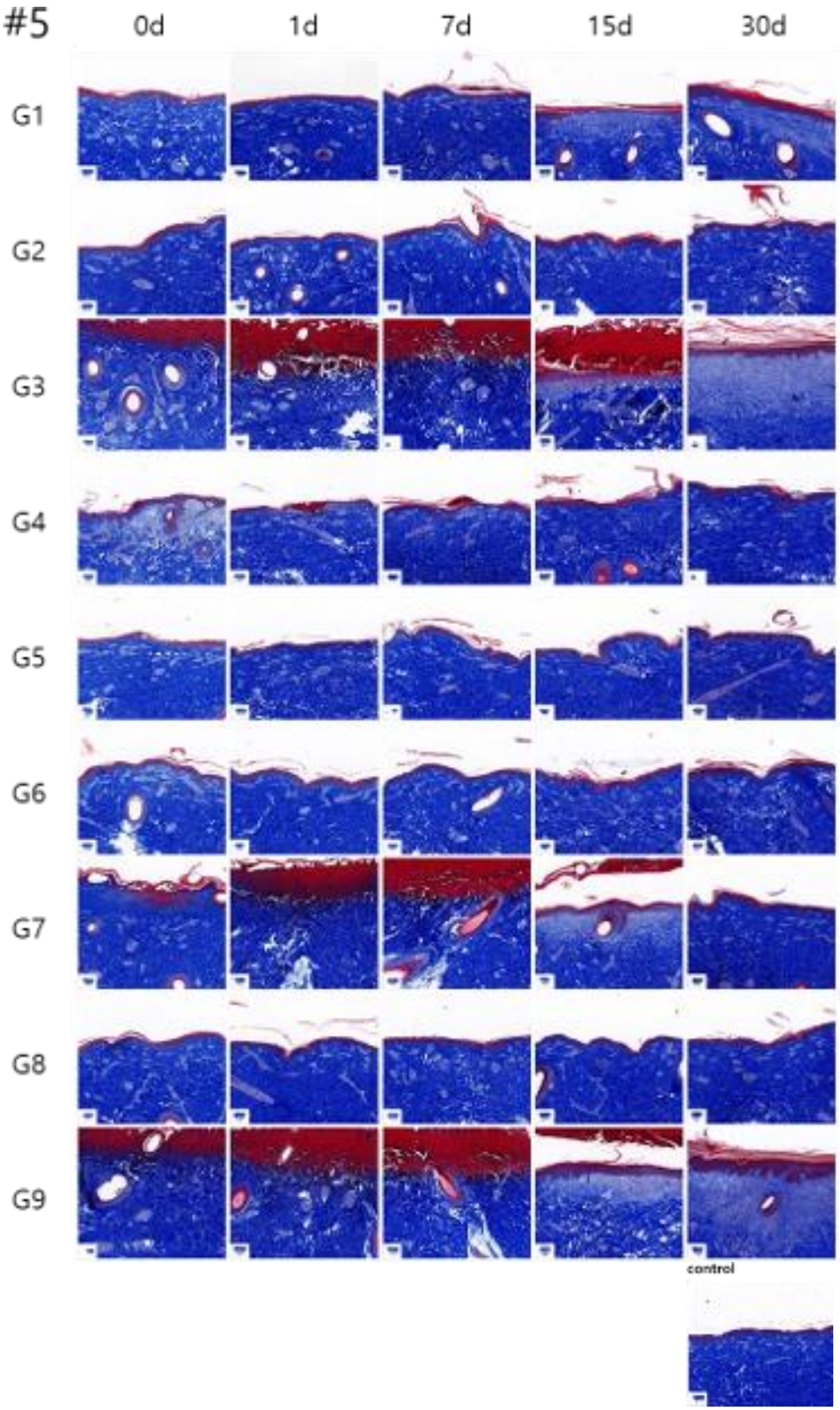
#5
0d
1d
7d
15d
30d
G1
G2
G3
G4
G5
G6
G7
G8
G9
control

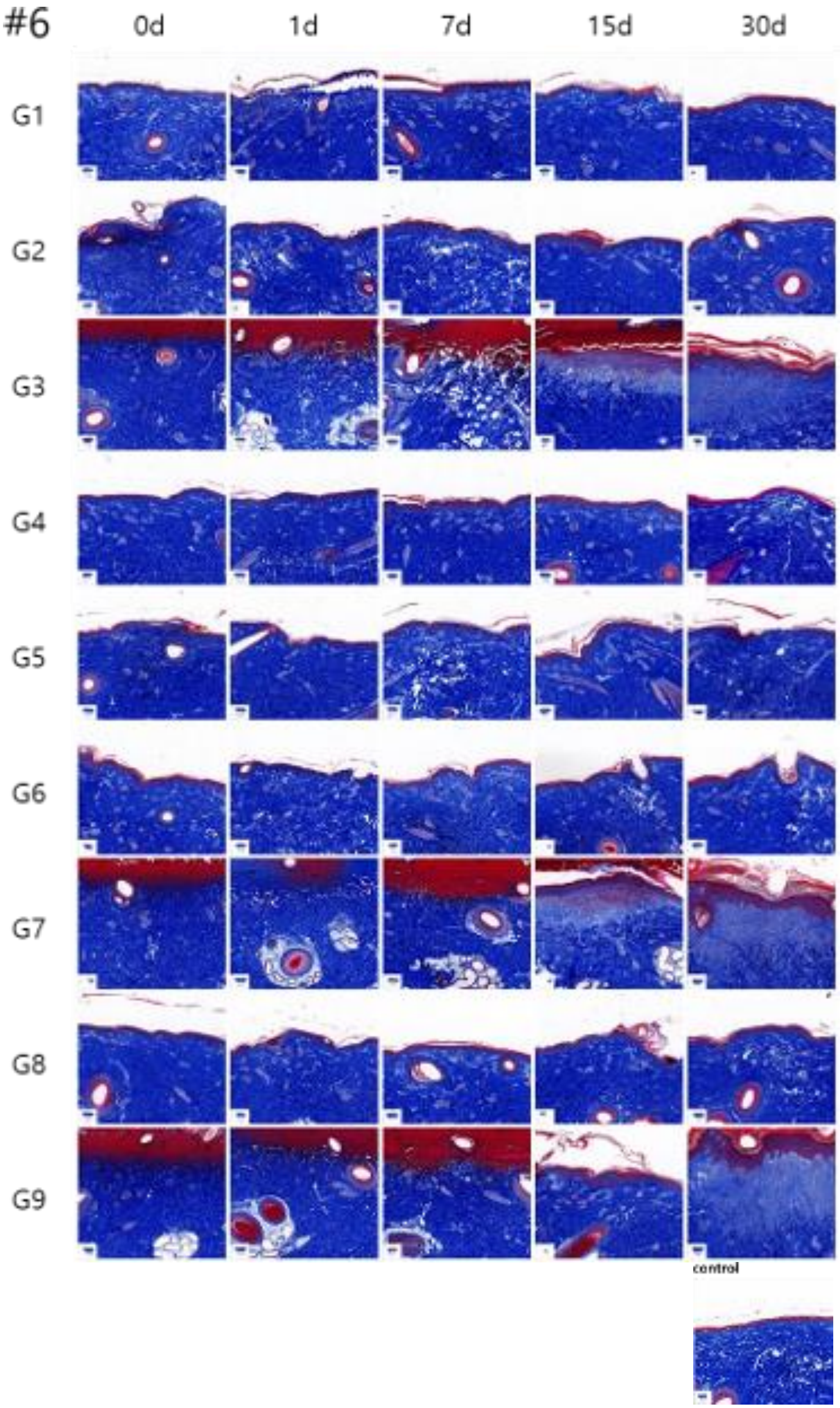
#6
0d
1d
7d
15d
30d
G1
G2
G3
G4
G5
G6
G7
G8
G9
control

Figure S5. Victoria blue staining images.

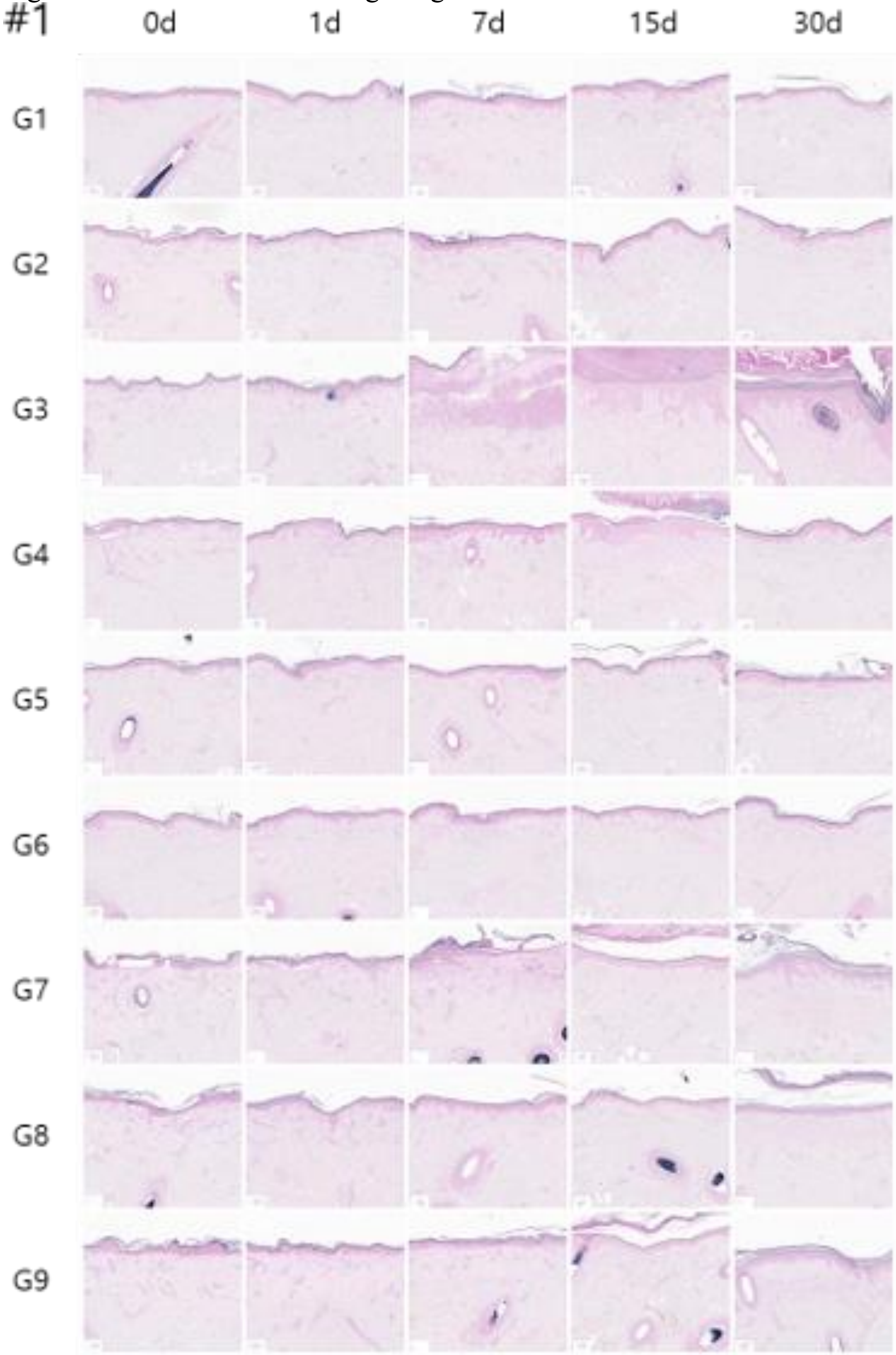

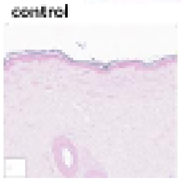

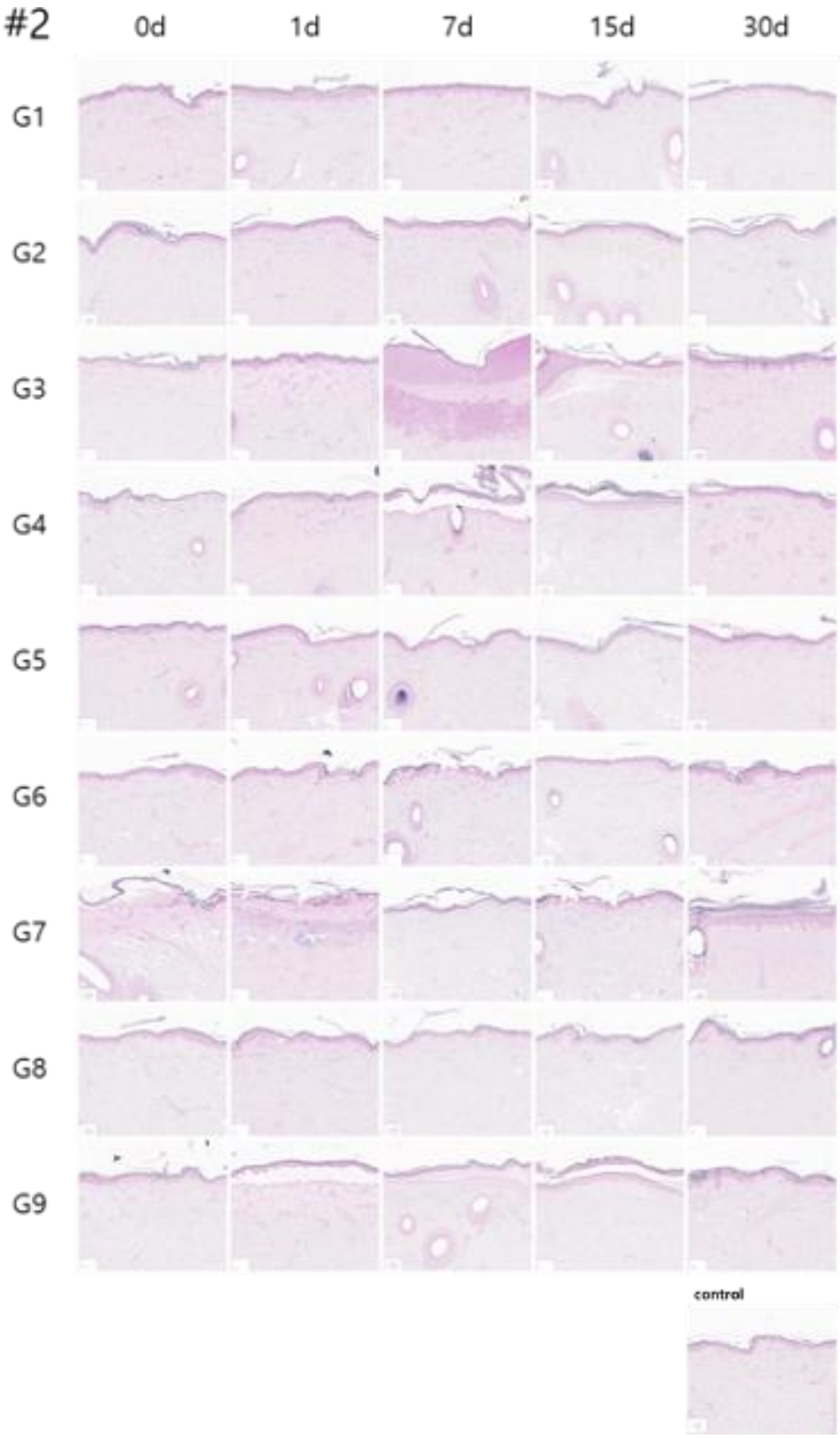
#2
0d
1d
7d
15d
30d
G1
G2
G3
G4
G5
G6
G7
G8
G9
control

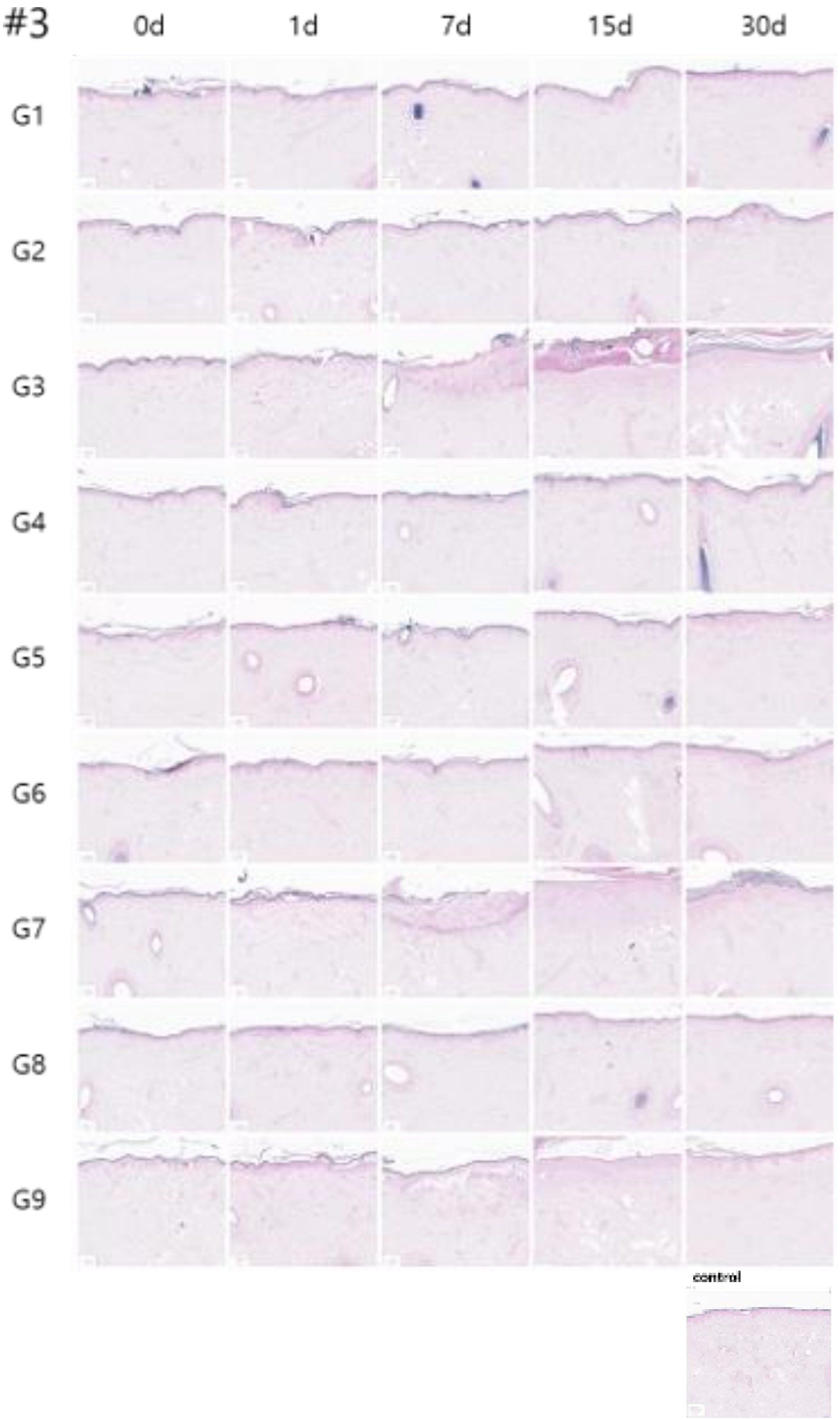
#3
0d
1d
7d
15d
30d
G1
G2
G3
G4
G5
G6
G7
G8
G9
control

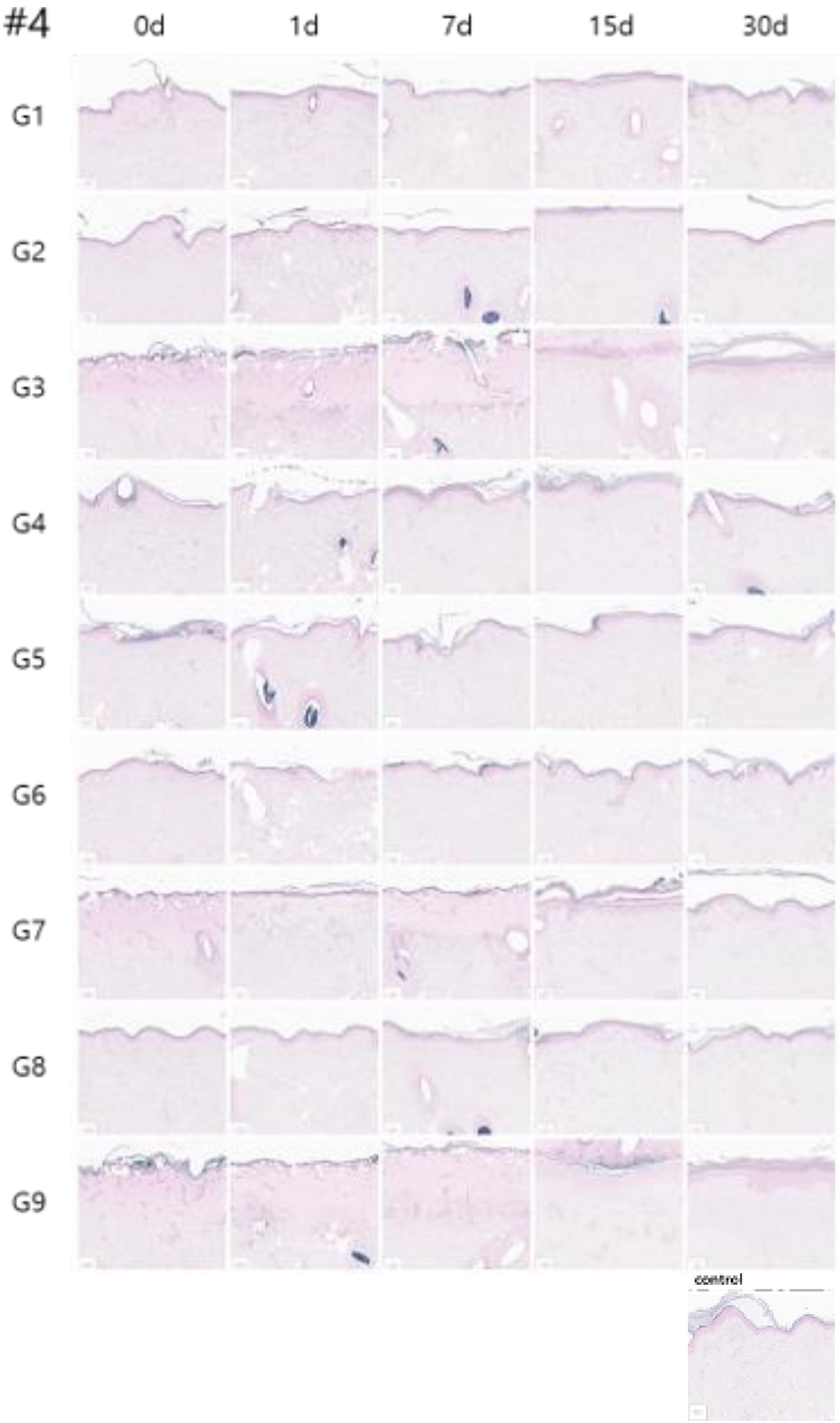
#4
0d
1d
7d
15d
30d
G1
G2
G3
G4
G5
G6
G7
G8
G9
control

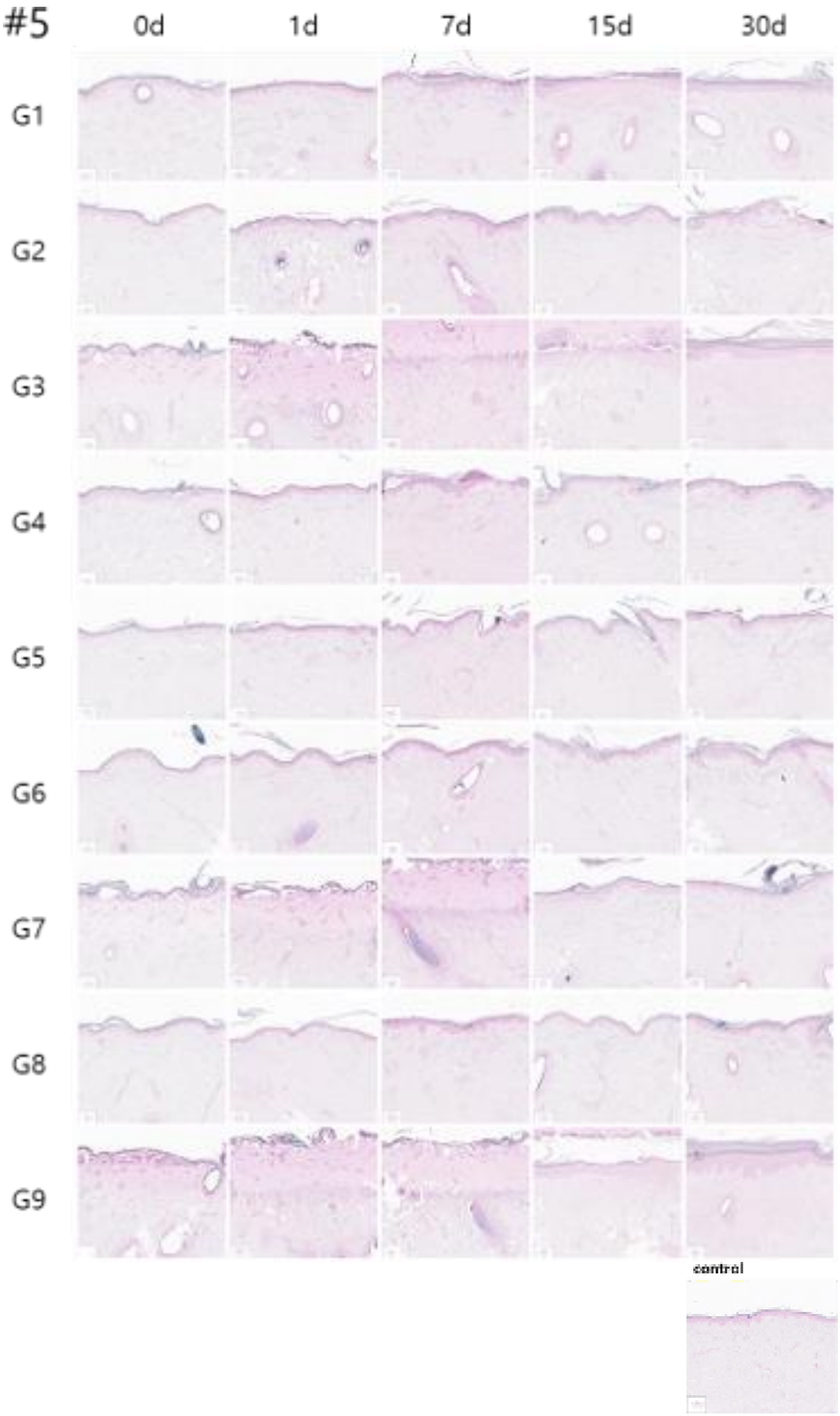

#5
0d
1d
7d
15d
30d
G1
G2
G3
G4
G5
G6
G7
G8
G9
control

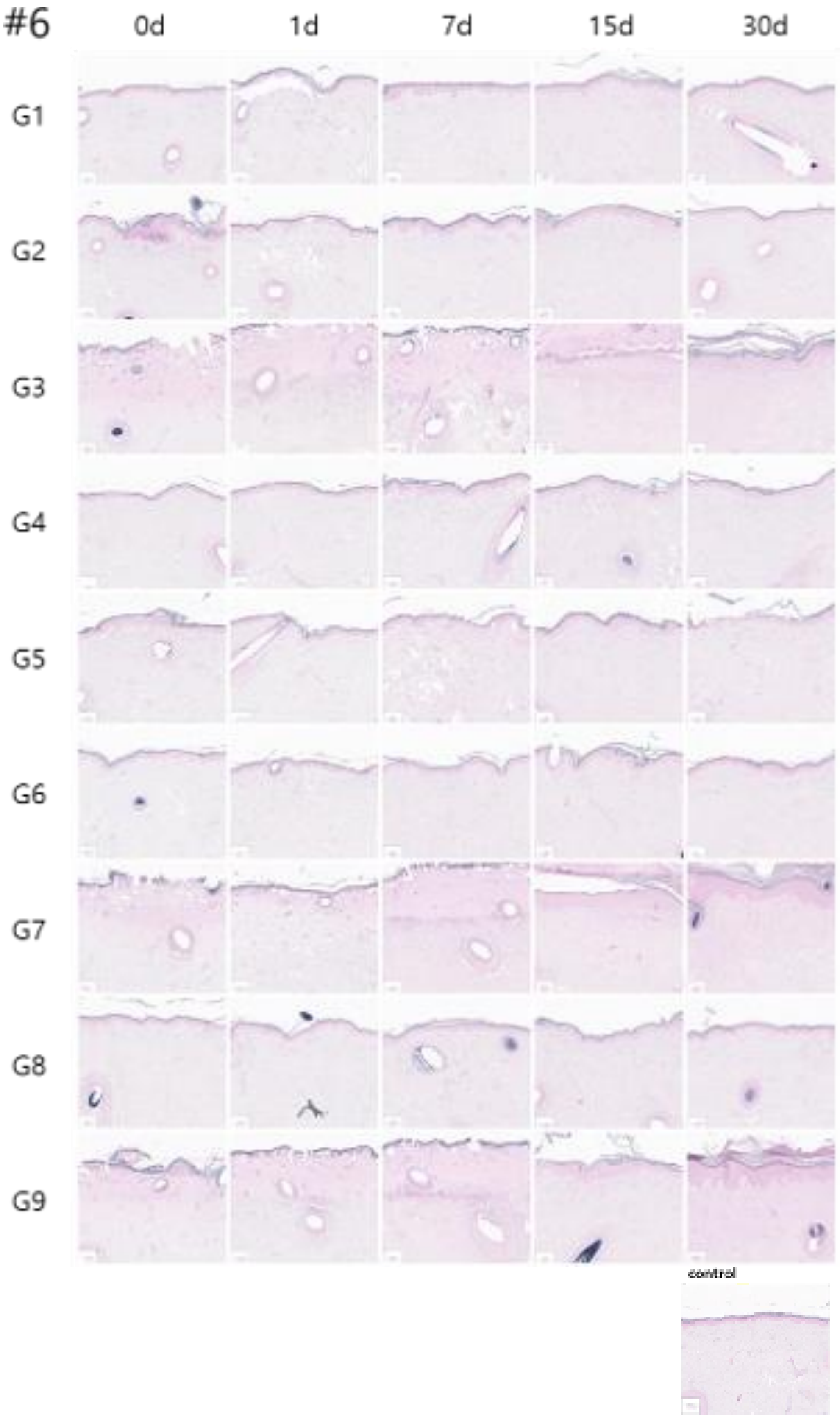
#6
0d
1d
7d
15d
30d
G1
G2
G3
G4
G5
G6
G7
G8
G9
control

Figure S6. NBTC staining images.

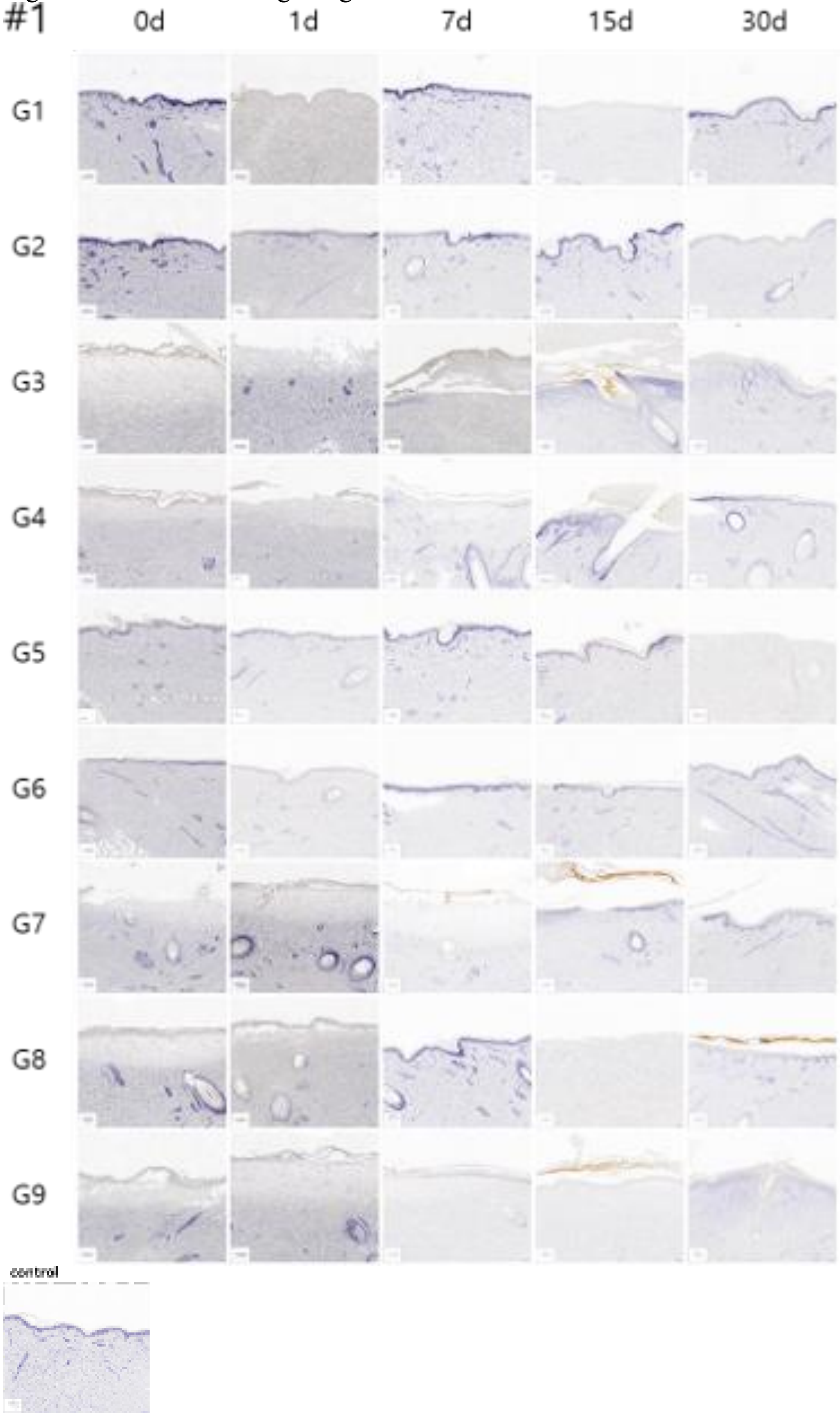

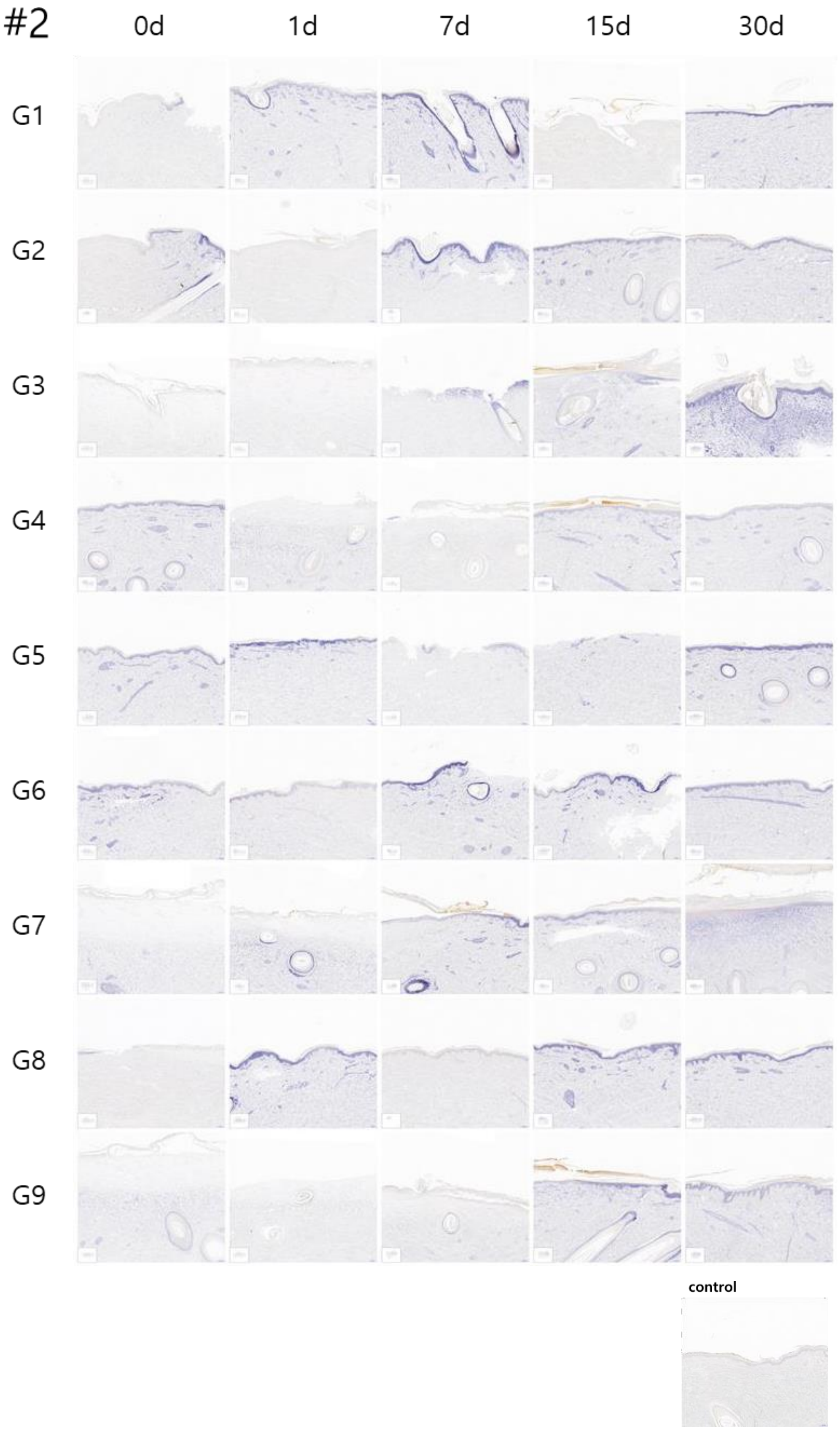
#2
0d
1d
7d
15d
30d
G1
G2
G3
G4
G5
G6
G7
G8
G9
control

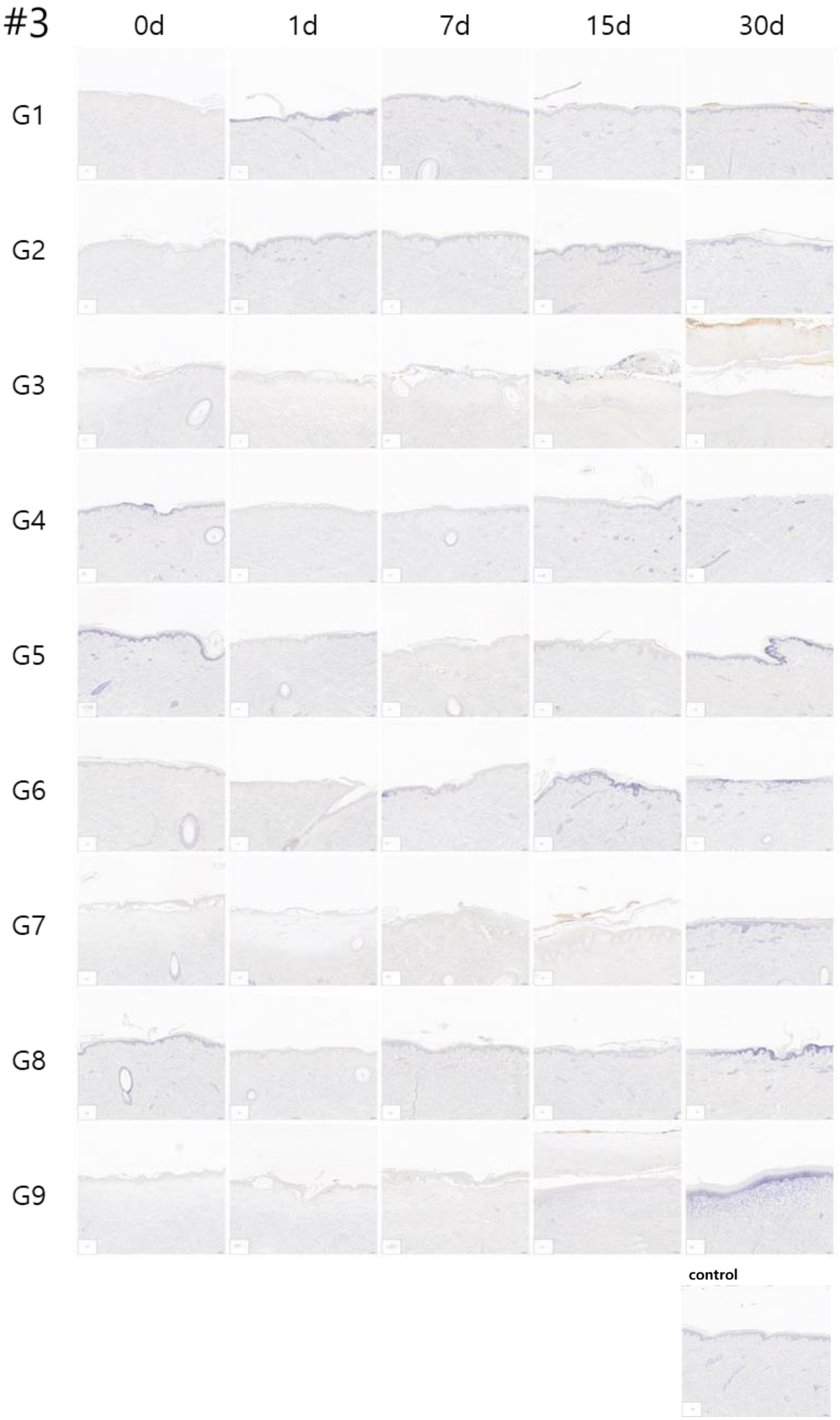
#3
0d
1d
7d
15d
30d
G1
G2
G3
G4
G5
G6
G7
G8
G9
control

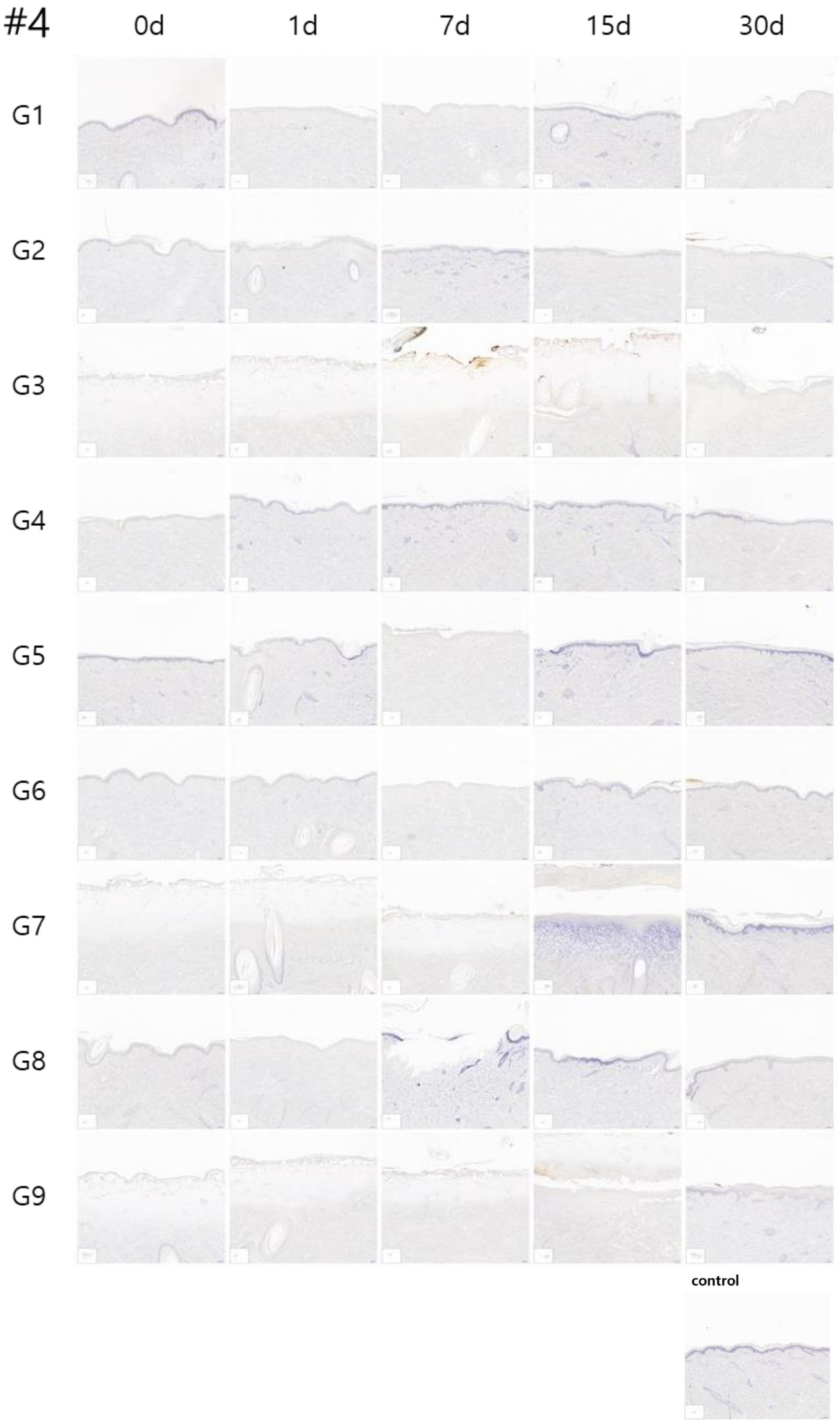
#4
0d
1d
7d
15d
30d
G1
G2
G3
G4
G5
G6
G7
G8
G9
control

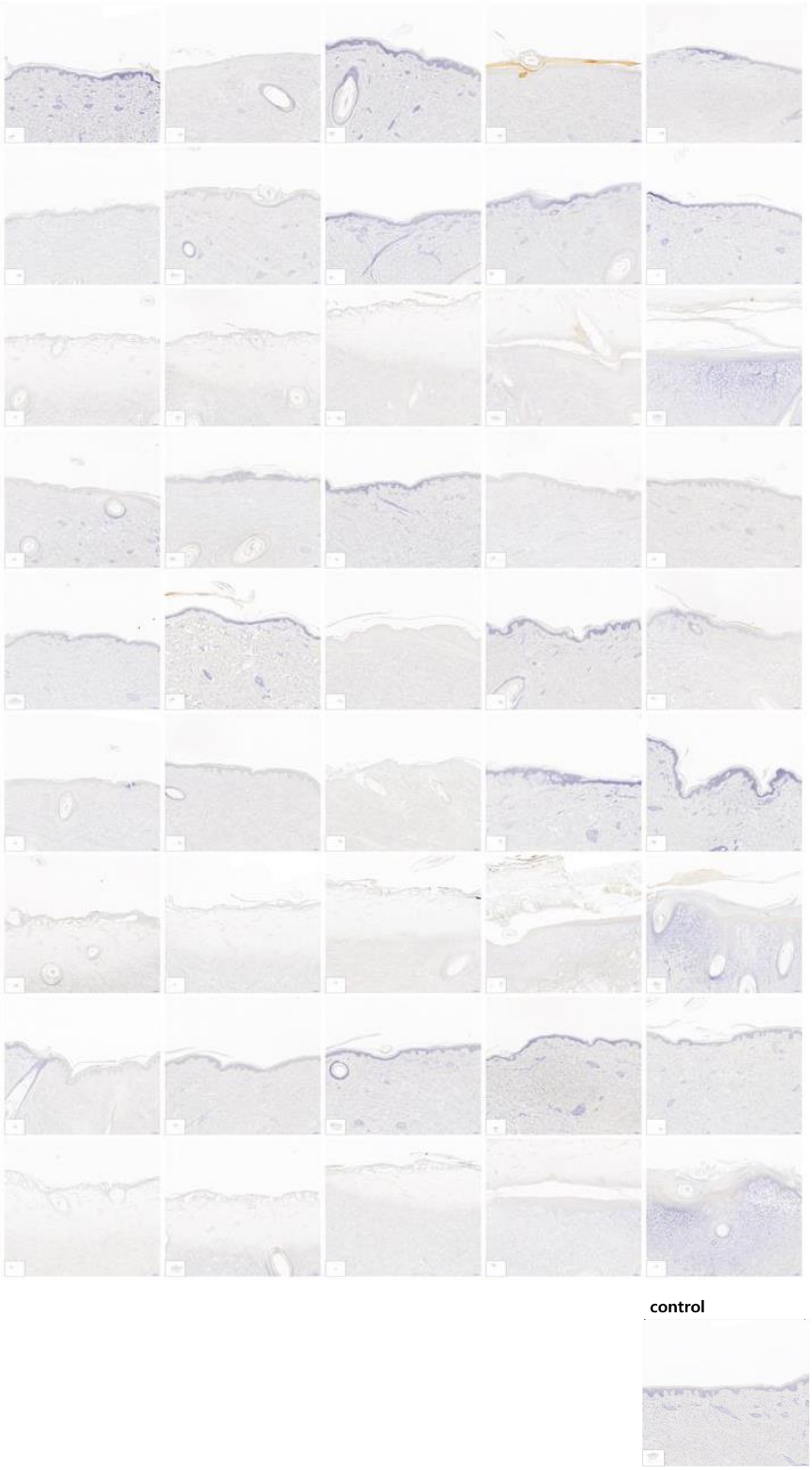
#5
0d
1d
7d
15d
30d
G1
G2
G3
G4
G5
G6
G7
G8
G9
control

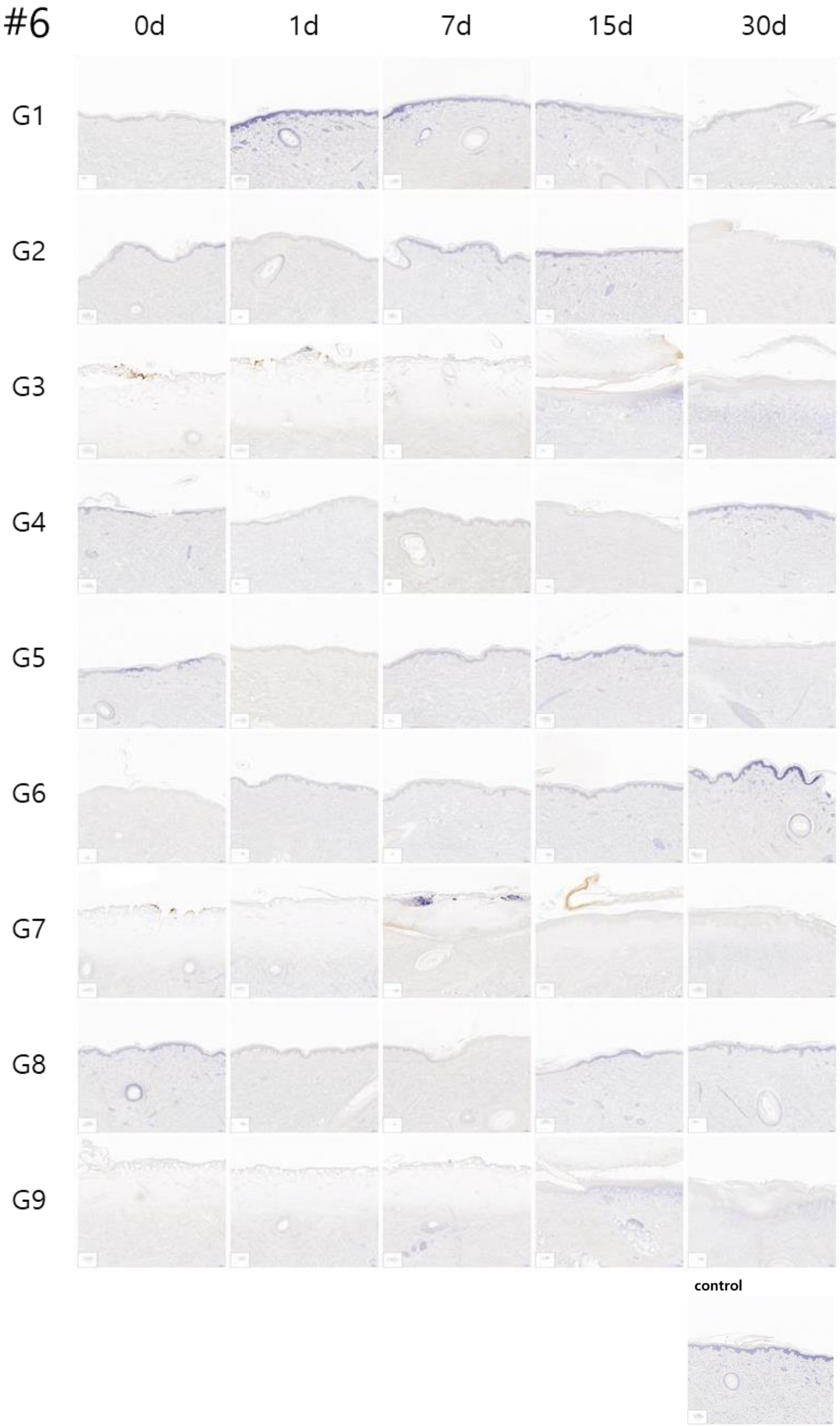
#6
0d
1d
7d
15d
30d
G1
G2
G3
G4
G5
G6
G7
G8
G9
control

TABLES

Table S1. Observation of animal body weight during the experiment period.

Gas resource	Animal	Day 0	Day 7	Day 14	Day 23	Day 30
He	#1	43.5	42.4	42.8	44.1	47.7
	#2	42.9	40.5	42	42.9	44.5
	#3	46.2	46.6	49.6	50.1	50.8
Ar	#4	39.7	40	40.9	43.8	46.6
	#5	40.5	41.9	43.8	45.2	47.6
	#6	40	40	42	44.6	47.2

Unit: Kilogram (Kg).

Table S2. The temperature of the skin surface immediately after the irradiation.

Gas	Day 0	G1	G2	G3	G4	G5	G6	G7	G8	G9
He	Mean	37.0	34.6	56.7	44.7	37.0	34.5	51.3	41.6	52.0
	SD	2.5	0.6	2.2	6.3	2.0	0.8	1.8	4.6	2.1
Ar	Mean	41.9	35.8	57.1	36.5	36.2	35.7	55.1	36.3	51.5
	SD	3.7	0.9	1.8	1.1	1.5	0.8	2.7	0.9	3.5

Values represent Mean ± S.D. Unit: °C

Table S3. Blood serum chemistry measurement at pre, 0, 1, 7, 15, 30 days of subjects using He gas.

He Gas	ALP (U/L)	AST (U/L)	ALT (U/L)	Ca (mg/dL)	IP (mg/dL)	BUN (mg/dL)	CRE (mg/dL)	TBIL (mg/dL)	TP (g/dL)	ALB (g/dL)	A/G ratio
Pre-Mean	131.0	25.9	26.2	10.3	9.5	4.5	1.2	0.14	6.3	3.0	0.9
S.D.	47.0	8.4	6.8	0.2	0.2	1.2	0.2	0.05	0.2	0.5	0.3
0d Mean	125.3	24.5	25.3	10.5	9.9	4.6	1.2	0.15	6.1	2.9	0.9
S.D.	44.3	7.9	6.5	0.4	0.7	1.0	0.2	0.09	0.3	0.5	0.3
1d-Mean	121.3	48.0	34.7	9.9	8.4	6.1	1.4	0.13	6.3	3.1	1.0
S.D.	27.2	15.8	7.0	0.3	0.9	2.5	0.2	0.06	0.2	0.5	0.3
7d-Mean	161.7	26.7	36.0	10.3	9.5	7.7	1.4	0.29	6.5	3.3	1.1
S.D.	49.0	13.6	5.8	0.1	0.9	2.5	0.2	0.19	0.3	0.4	0.2
15d-Mean	139.7	18.8	34.6	10.5	7.6	6.3	1.4	0.08	6.5	3.2	1.0
S.D.	42.3	7.0	1.8	0.2	1.0	0.3	0.0	0.01	0.2	0.2	0.1
30d-Mean	149.3	23.5	37.5	9.9	8.4	6.0	1.7	0.14	6.3	3.2	1.0
S.D.	68.2	6.1	6.2	0.2	0.5	0.8	0.3	0.06	0.3	0.1	0.2

Values represent Mean ± S.D.

Table S4. Blood serum chemistry measurement at pre, 0, 1, 7, 15, 30 days of subjects using Ar gas.

Argon Gas	ALP (U/L)	AST (U/L)	ALT (U/L)	Ca (mg/dL)	IP (mg/dL)	BUN (mg/dL)	CRE (mg/dL)	TBIL (mg/dL)	TP (g/dL)	ALB (g/dL)	A/G ratio
Pre-Mean	148.3	32.7	30.3	10.1	9.4	4.8	1.6	0.14	6.7	3.1	0.9
S.D.	54.8	10.2	4.9	0.2	0.1	2.4	0.2	0.06	0.7	0.2	0.1
0d Mean	142.7	32.4	29.5	10.1	10.2	4.9	1.5	0.13	6.6	3.0	0.9
S.D.	53.5	13.7	4.8	0.5	0.3	2.0	0.2	0.05	0.7	0.1	0.1
1d-Mean	140.3	56.8	39.1	9.7	7.7	6.3	1.6	0.10	6.8	3.2	0.9
S.D.	49.2	28.8	3.6	0.4	0.2	1.0	0.1	0.01	0.7	0.1	0.2
7d-Mean	142.0	27.2	30.4	10.1	8.4	5.2	1.5	0.18	6.7	3.2	1.0
S.D.	55.8	16.7	6.5	0.4	0.4	1.1	0.2	0.08	0.3	0.1	0.1
15d-Mean	149.7	23.5	32.0	10.9	6.4	7.5	1.6	0.07	6.8	3.2	0.9
S.D.	73.2	12.1	4.9	0.4	1.1	1.7	0.3	0.01	0.5	0.2	0.2
30d-Mean	149.0	23.1	30.8	10.3	8.3	9.3	1.9	0.06	6.6	3.3	1.0
S.D.	89.0	3.5	5.7	1.1	1.0	2.7	0.4	0.01	0.0	0.4	0.2

Values represent *Mean* ± *S.D.*

Table S5. Thickness of the epithelial tissue in the application site (μm).

Gas	Group	Day	AVG	STDEV	n
He Gas	G1	15d	50.64	10.34	9
		30d	42.71	9.66	9
	G2	15d	52.13	9.18	9
		30d	48.39	9.31	9
	G3	15d	115.29	33.61	9
		30d	111.02	47.30	9
	G4	15d	72.31	25.87	9
		30d	54.00	9.44	9
	G5	15d	52.34	10.46	9
		30d	45.94	10.12	9
	G6	15d	41.99	2.36	9
		30d	48.88	8.10	9
	G7	15d	106.51	40.32	9
		30d	100.66	62.38	9
	G8	15d	47.04	6.97	9
		30d	52.79	10.79	9
	G9	15d	99.10	47.57	9
		30d	70.37	15.70	9
	Control		49.92	12.21	9
Ar Gas	G1	15d	51.18	10.73	9
		30d	58.07	20.16	9
	G2	15d	68.07	45.23	9
		30d	49.61	14.82	9
	G3	15d	181.16	51.31	9
		30d	125.47	23.57	9
	G4	15d	56.99	11.20	9
		30d	47.37	10.62	9
	G5	15d	53.78	8.15	9
		30d	59.16	15.53	9
	G6	15d	50.17	8.09	9

	30d	48.68	8.12	9
G7	15d	128.43	38.74	9
	30d	103.26	88.26	9
G8	15d	50.68	7.73	9
	30d	48.50	6.53	9
G9	15d	120.91	25.60	9
	30d	133.29	32.60	9
Control		52.33	14.65	9

Table S6. Collagen deposition rate at the application site (%). Collagen density was evaluated using maason's trichrome staining.

Gas	Group	Day	AVG	STDEV	n
He Gas	G1	15d	68.96	3.37	3
		30d	67.75	6.57	3
	G2	15d	62.94	4.38	3
		30d	64.56	5.98	3
	G3	15d	32.60	14.21	3
		30d	39.05	20.05	3
	G4	15d	45.22	14.71	3
		30d	57.67	15.40	3
	G5	15d	64.82	15.97	3
		30d	69.89	5.55	3
	G6	15d	65.97	4.43	3
		30d	70.28	13.62	3
	G7	15d	25.69	17.65	3
		30d	32.03	9.60	3
	G8	15d	60.79	5.17	3
		30d	58.75	15.04	3
	G9	15d	30.65	12.95	3
		30d	45.06	4.66	3
	Control		70.55	10.27	3
Ar Gas	G1	15d	33.92	16.03	3
		30d	54.05	14.88	3
	G2	15d	59.64	2.12	3
		30d	59.44	5.62	3
	G3	15d	16.47	6.82	3
		30d	19.30	6.35	3
	G4	15d	56.58	3.10	3
		30d	60.66	2.36	3
	G5	15d	55.32	8.87	3
		30d	54.73	4.39	3
	G6	15d	62.23	6.19	3

	30d	54.32	3.90	3
G7	15d	22.17	12.21	3
	30d	40.86	27.95	3
G8	15d	67.08	3.27	3
	30d	61.18	4.90	3
G9	15d	26.55	17.28	3
	30d	22.03	4.19	3
Control		56.19	8.44	3

Table S7. Changes in the proportion of elastic fibers at the application site (%). Area of elastic fiber was evaluated using Victoria blue staining.

Gas	Group	Day	AVG	STDEV	n
He Gas	G1	15d	2.94	0.67	3
		30d	3.14	0.82	3
	G2	15d	2.29	0.12	3
		30d	3.06	0.89	3
	G3	15d	0.90	0.37	3
		30d	1.07	0.38	3
	G4	15d	2.12	1.43	3
		30d	2.40	1.30	3
	G5	15d	2.98	0.75	3
		30d	2.46	0.66	3
	G6	15d	2.79	0.58	3
		30d	2.08	0.23	3
	G7	15d	1.27	0.26	3
		30d	1.43	0.08	3
	G8	15d	2.95	0.82	3
		30d	1.99	0.47	3
	G9	15d	1.03	0.46	3
		30d	1.77	0.84	3
	Control		2.26	0.65	3
Ar Gas	G1	15d	1.88	0.50	3
		30d	2.76	0.72	3
	G2	15d	2.78	0.66	3
		30d	3.35	0.70	3
	G3	15d	1.00	0.56	3
		30d	1.43	0.27	3
	G4	15d	2.98	0.79	3
		30d	3.68	0.19	3
	G5	15d	3.05	0.87	3
		30d	3.47	0.65	3
	G6	15d	3.38	0.78	3

		30d	3.42	1.06	3
	G7	15d	1.65	1.32	3
		30d	2.98	2.37	3
	G8	15d	3.48	0.71	3
		30d	3.77	0.82	3
	G9	15d	1.49	0.69	3
		30d	1.11	0.68	3
	Control		3.44	0.81	3

Table S8. Plasma irradiation induced coagulation area (mm^2). The coagulation area was evaluated using NBTC staining.

Gas	Group	Day	AVG	STDEV	n
He Gas	G1	0d	0	0	3
		1d	0	0	3
	G2	0d	0	0	3
		1d	0	0	3
	G3	0d	7.05	3.21	3
		1d	5.31	1.21	3
	G4	0d	1.54	1.37	3
		1d	1.35	1.18	3
	G5	0d	0	0	3
		1d	0	0	3
	G6	0d	0	0	3
		1d	0	0	3
	G7	0d	4.49	0.73	3
		1d	3.62	0.62	3
	G8	0d	1.60	1.39	3
		1d	0.66	0.59	3
	G9	0d	4.07	0.73	3
		1d	3.40	2.36	3
	Control		0	0	3
Ar Gas	G1	0d	0	0	3
		1d	0	0	3
	G2	0d	0	0	3
		1d	0	0	3
	G3	0d	9.52	2.64	3
		1d	9.30	1.06	3
	G4	0d	0	0	3
		1d	0	0	3
	G5	0d	0	0	3
		1d	0	0	3
	G6	0d	0	0	3

		1d	0	0	3
	G7	0d	5.68	0.90	3
		1d	5.39	0.52	3
	G8	0d	0	0	3
		1d	0	0	3
	G9	0d	5.35	0.29	3
		1d	5.98	0.32	3
Control			0	0	3
